# Fine-Tuning of the Kaposi’s Sarcoma-Associated Herpesvirus Life Cycle in Neighboring Cells through the RTA-JAG1-Notch Pathway

**DOI:** 10.1371/journal.ppat.1005900

**Published:** 2016-10-19

**Authors:** Shasha Li, Hao Hu, Zhiheng He, Deguang Liang, Rui Sun, Ke Lan

**Affiliations:** Key Laboratory of Molecular Virology and Immunology, Institut Pasteur of Shanghai, Chinese Academy of Sciences, Shanghai, China; Tulane Health Sciences Center, UNITED STATES

## Abstract

Kaposi’s sarcoma (KS)-associated herpesvirus (KSHV) is an oncogenic pathogen that displays latent and lytic life cycles. In KS lesions, infiltrated immune cells, secreted viral and/or cellular cytokines, and hypoxia orchestrate a chronic pro-lytic microenvironment that can promote KSHV reactivation. However, only a small subset of viruses spontaneously undergoes lytic replication in this pro-lytic microenvironment while the majority remains in latency. Here, we show that the expression of the Notch ligand JAG1 is induced by KSHV-encoded replication and transcription activator (RTA) during reactivation. JAG1 up-regulation activates Notch signaling in neighboring cells and prevents viral lytic replication. The suppression of JAG1 and Notch1 with inhibitors or small interfering RNA promotes lytic replication in the presence of RTA induction or under conditions of hypoxia. The underlying mechanism involves the Notch downstream effector hairy and enhancer of split 1 (Hes1), which directly binds lytic gene promoters and attenuates viral lytic gene expression. RTA interacts with lymphoid enhancer-binding factor 1 (LEF1), disrupts LEF1/Groucho/TLE suppressive complexes and releases LEF1 to activate JAG1 expression. Taken together, our results suggest that cells with viral lytic replication can inhibit KSHV reactivation in neighboring cells through an RTA-JAG1-Notch pathway. These data provide insight into the mechanism by which the virus maintains the balance between lytic and latent infection in the pro-lytic tumor microenvironment.

## Author Summary

KSHV infected cells display significant heterogeneity in viral lytic replication within the universal pro-lytic inflammatory milieu, suggesting that the balance between latency and reactivation is carefully regulated. This fine-tuned regulatory mechanism is essential for KSHV to persist in the host and drive cells to malignancy. In the present study, we show that KSHV can usurp the Notch signaling pathway to inhibit the viral lytic life cycle in neighboring cells. Notch signaling in surrounding cells can be activated through an RTA-JAG1-Notch pathway initiated by cells in which KSHV is reactivated. Activated Notch inhibits KSHV reactivation through its downstream effector Hes1. These findings suggest that the ability of Notch to determine the fate of adjacent cells is hijacked by KSHV to maintain its life cycle, providing a mechanistic explanation for the phenomenon by which only a small fraction of viruses enters lytic replication in the common pro-lytic microenvironment.

## Introduction

Kaposi’s sarcoma (KS)-associated herpesvirus (KSHV) is a large double-stranded DNA virus with a biphasic life cycle [1]. In KS lesions, KSHV latently infects most tumor cells to maintain viral DNA [2, 3], evade host immunosurveillance [4], and promote cellular proliferation [5]. The viruses in a small subset of infected cells spontaneously switch into the lytic replication cycle from latency, expressing viral lytic products such as replication and transcription activator (RTA), open reading frame K8 (K-bZIP), human herpesvirus 8 interleukin-6 (vIL6), open reading frame 45 (ORF45) and open reading frame 59 (ORF59) [6–9]. The lytic viruses may benefit KS pathogenesis by re-infecting the neighboring cells [10] and releasing pro-inflammatory or angiogenic cytokines in a paracrine manner [9].

Previous studies suggest that extrinsic factors such as hypoxia [11–14], oxidative stress [15, 16], and inflammation [17, 18], can trigger the change from latency to lytic replication in viruses. Furthermore, KS tissues perfuse with slit-like vessels and a large number of infiltrated inflammatory cells exhibit a pro-lytic milieu that potentially promotes KSHV to be reactivated from latency [19]. The latently infected cells are likely to become stressed, and the virus can be stimulated to undergo lytic replication associated with tumor progression. However, reactivation is a rare event in KS tissues, with approximately 1–3% of spindle cells displaying lytic replicative markers [19]. In this regard, the controlled lytic replication observed in KS tissues suggests its pathological importance for disease development. However, the mechanism by which KSHV regulates this process remains unclear.

The Notch signaling pathway is critical for KS development. The Notch ligands JAG1 and Dll4, and the Notch receptors Notch1–4 are highly expressed in KS tumor cells [20]. Notch signaling is evolutionarily conserved in most multicellular organisms. It enables short-range communication between the cells of metazoans through physical contact [21] and regulates many cellular functions including proliferation, death, and differentiation [21–24]. It is unique for its ability to specify the fate of the adjacent cells within an equivalence group into different (sometimes opposite) directions by cell-to-cell communication and subsequently altered gene expression, known as lateral inhibition [25–27]. Aberrant gain or loss of Notch function is linked to a wide range of human disorders, including developmental disorders and cancers [28, 29].

Based on these data, we hypothesized that Notch may specify the fate of viruses in infected cells. In the present study, we found that KSHV RTA up-regulates the Notch ligand JAG1 by interacting with LEF1 and triggers Notch activation in neighboring cells. The activated Notch inhibits KSHV reactivation in those neighboring cells. We found that the inhibitory effect of Notch on KSHV reactivation largely relies on the HES/HEY family, which could directly bind and inhibit a number of major KSHV lytic gene promoters. Our study describes a novel regulatory process by which KSHV specifies latency and reactivation in virus-infected cells. Our findings provide insight into the mechanism by which a minority of viruses undergoes reactivation, while the majority maintains a persistent latent infection in KS tissues.

## Results

### KSHV RTA up-regulates JAG1 and activates the Notch signaling pathway

**Fig 1:**
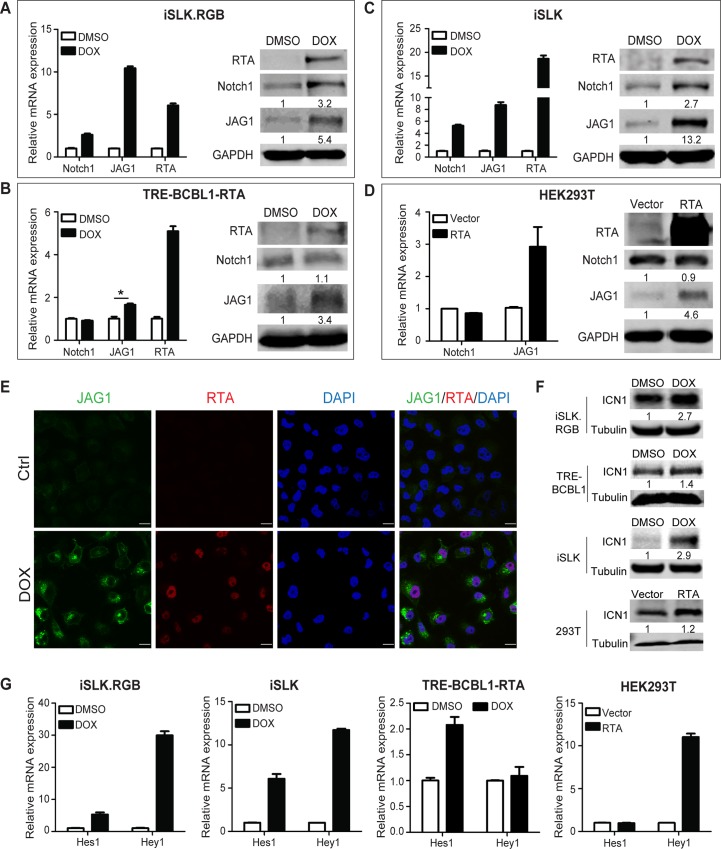
RTA up-regulates Notch components in different cell types. (A–C) iSLK.RGB cells, TRE-BCBL1-RTA cells, and iSLK cells were plated in 6-well plates at 0.1 million cells per well. After 24 h, the cells were induced with doxycycline (5 μg/ml) for 24 h and the expression of Notch components was measured by qPCR and western blotting. The data were normalized to GAPDH expression. (D) HEK293T cells seeded in 6-well plates were transfected with RTA (4 μg) using Lipofectamine 2000 for 24 h and the expression of Notch components was measured by qPCR and western blotting. (E) Immunofluorescence imaging of iSLK cells treated with or without doxycycline for 24 h. The JAG1 (Green) and RTA (Red) in the nucleus were labeled with the indicated primary and secondary antibodies. Scale bars represent 20 μm. (F) iSLK.RGB cells, TRE-BCBL1-RTA cells and iSLK cells were plated in 6-well plates at 0.1 million cells per well and treated with or without doxycycline for 24 h. HEK293T cells were transfected with RTA (4 μg) using Lipofectamine 2000 for 24 h in 6-well plates. The protein level of ICN1 was quantified by western blotting. (G) Hes1 and Hey1 were quantified by qPCR. The data were normalized to GAPDH expression. Data were expressed as the mean ± s.e.m., n = 3, *p<0.05.

RTA is the master switch molecule that drives the lytic reactivation of KSHV from latency. Previous studies have shown that RTA usurps Notch downstream transcription factor RBP-Jκ to activate the lytic genes during reactivation [30–32]. However, how RTA or KSHV lytic reactivation modulates Notch signaling pathway remains largely unknown. In the present study, RTA was induced to initiate lytic reactivation in iSLK.RGB (iSLK cells stably infected with a novel reporter KSHV virus called red-green-blue-BAC16 [33]) and TRE-BCBL1-RTA cells [34] upon doxycycline treatment. Assessment of the expression of the core components of the Notch signaling pathway showed a significant up-regulation of Notch1 and JAG1 at mRNA and protein levels in iSLK.RGB cells (Figs 1A and S1A). JAG1 was also up-regulated in the TRE-BCBL1-RTA cell line, whereas Notch1 levels remained relatively unchanged (Fig 1B).

To evaluate the effect of RTA on the activity of the Notch pathway, the iSLK cell line, which contains an inducible RTA cassette but lacks the KSHV episome, was treated with doxycycline to avoid the noise from other KSHV genes. Induction of RTA significantly up-regulated JAG1 and Notch1 at mRNA and protein level (Fig 1C). Notch ligands and receptors remained unchanged upon doxycycline stimulation in SLK cells, which lack the RTA expression cassette (S1B Fig). This eliminated the artifacts related to drug treatment and suggested that RTA plays a role in Notch regulation. Ectopic expression of RTA in HEK293T cells resulted in the up-regulation of JAG1, whereas Notch1 remained unchanged compared with the control counterparts (Fig 1D). Immunofluorescence staining showed facilitated JAG1 protein synthesis and JAG1 was accumulated on the cell membrane without external secretion in doxycycline treated iSLK cells compared with the pattern in untreated cells (Fig 1E). Approximately 90 percent of the cells overexpressing RTA showed up-regulation of JAG1 at protein level (433 out of 477 cells) in the immunofluorescence images at lower magnitude (S1C Fig).

To determine whether the up-regulation of JAG1 and Notch1 contributed to Notch pathway activation, the generation of Notch Intracellular domain (ICN) and expression of the downstream effectors of the Notch pathway, including Hes1 and hairy/enhancer-of-split related with YRPW motif protein 1 (Hey1), were assessed. The production of ICN1 was elevated with RTA expression in above mentioned four cell lines, even though Notch1 receptor was not up-regulated in either TRE-BCBL1 or HEK293T cells (Fig 1F). With regard to the Notch downstream targets, results showed a significant up-regulation of Hes1 and Hey1 in iSLK cells with or without KSHV infection upon doxycycline treatment (Fig 1G). However, in TRE-BCBL1-RTA cells, Hes1 was up-regulated while Hey1 levels remained unchanged (Fig 1G). Overexpression of RTA in HEK293T cells only led to Hey1 up-regulation (Fig 1G). Taken together, these results demonstrated that Notch components, especially the Notch ligand JAG1, were up-regulated in cells with KSHV RTA expression, resulting in the activation of the Notch pathway.

### JAG1 in RTA-expressing cells activates the Notch signaling pathway in neighboring cells

**Fig 2:**
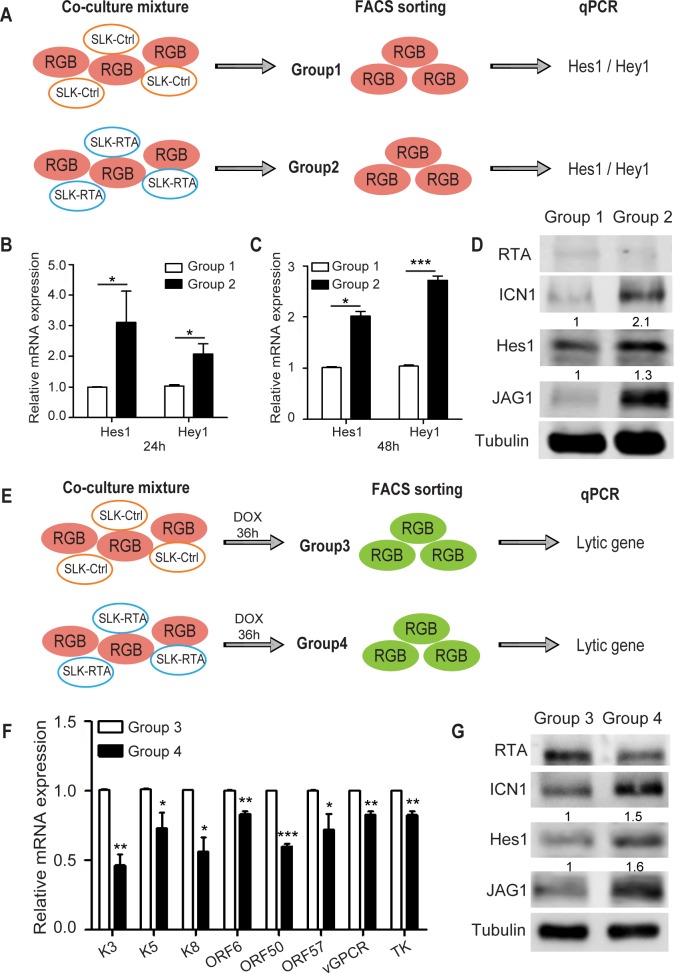
RTA induced JAG1 activates Notch signaling in neighboring cells through direct interaction. (A) Schematic illustrating the co-culture assay. SLK-Ctrl cells (0.4 million cells) or SLK-RTA cells (0.4 million cells) were co-cultured with iSLK.RGB (0.4 million cells) in 100 mm dish. At 24 and 48 h post co-culture, the RFP positive iSLK.RGB cells were sorted by FACS, followed by quantification of Hes1 and Hey1 expression by qPCR (B, C) or RTA, ICN1, Hes1 and JAG1 expression by western blotting (D). (E) Schematic illustrating the co-culture assays to detect lytic gene expression. SLK-Ctrl cells (0.4 million cells) or SLK-RTA cells (0.4 million cells) were co-cultured with iSLK.RGB (0.4 million cells) in 100 mm dish for 24 h and cells were treated with a low dose of doxycycline (100 ng/ml) to induce reactivation for 36 h. RFP and GFP double positive iSLK.RGB cells were sorted by FACS, followed by quantification of lytic gene expression by qPCR (F) or RTA, ICN1, Hes1 and JAG1 expression by western blotting (G). Data were expressed as the mean ± s.e.m., n = 3, *p<0.05, **p<0.01, ***p<0.001.

As Notch activation is initiated by ligand-receptor interaction through cell-to-cell contact, we examined whether RTA induced JAG1 could activate Notch signaling in neighboring cells *in trans*. For this purpose, we established a co-culture system to mimic KSHV infection status in KS lesions by pooling RTA-expressing cells and KSHV latently infected cells. A new SLK cell line was established to ectopically express RTA (SLK-RTA) as a Notch signaling donor. Consistently, JAG1 was up-regulated in SLK-RTA cells compared with control cells (SLK-Ctrl) (S2A Fig). iSLK.RGB cells, as the Notch signaling recipient, were co-cultured with SLK-Ctrl or SLK-RTA cells for 24 and 48 h. Then, iSLK.RGB cells were sorted by Flow Cytometry (FACS) based on RFP expression as group1 and group2 respectively (S2G Fig) and Notch activation status was tested by detecting Hes1/Hey1 and intracellular Notch 1 (ICN1) expression (Fig 2A). The results showed that Hes1 and Hey1 were significantly up-regulated in iSLK.RGB cells after 24 or 48 h in group2 compared with those in group1 (Fig 2B and 2C), although Hey1 was expressed at a relatively lower level after 24 h. iSLK.RGB cells co-cultured with SLK-RTA cells increased endogenous ICN1 and Hes1 protein levels, whereas ICN1 and Hes1 production in group1 remained at basal levels (Fig 2D). The expression of RTA was undetectable but we observed an elevated expression of JAG1, which might be due to Notch activation (Fig 2D) [35, 36]. This process was contact dependent, as Notch activation did not occur when the cells were co-cultured using a Transwell membrane to avoid cell-to-cell contact (S2B Fig). These data demonstrated that the expression of JAG1 in RTA-expressing cells can induce Notch activation in adjacent cells.

We next examined whether Notch activation affects KSHV lytic reactivation in neighboring cells by co-culturing doxycycline-induced iSLK.RGB cells with SLK-Ctrl or SLK-RTA cells. As SLK cells lack doxycycline responsive elements, treatment with doxycycline should induce RTA only in iSLK.RGB cells. The two co-culture mixtures were treated with low concentrations of doxycycline for 36 h, approximately 13% of RFP and GFP double positive iSLK.RGB cells (cells with viral lytic reactivation) were isolated as group3 and group4 respectively (S2H Fig) for qPCR and western blotting analysis (Fig 2E). Representative KSHV lytic genes were selected for quantification based on their expression at different stages during reactivation process, including RTA, K5, and ORF57 as immediate early genes; K3 and K8 as early lytic genes; and ORF6, KSHV G protein-coupled receptor (vGPCR), and KSHV thymidine kinase (TK) as late lytic genes [37]. To differentiate the RTA transcripts expressed by the KSHV genome and those induced by the doxycycline inducible cassette, primers specific for the 3′-UTR of RTA, which only amplified RTA transcript expressed by KSHV, were designed (S2 Table). iSLK.RGB cells (group4) with Notch activation induced by SLK-RTA displayed a repressed KSHV lytic gene expression pattern compared with that in group3 (Fig 2F). Despite the potential induction of noise by iSLK.RGB itself, which may interfere with the assessment of Notch activation, increased Notch activity (the higher level of ICN1 and Hes1) was observed in cells from the SLK-RTA co-culture group (Fig 2G) and was negatively correlated with KSHV lytic reactivation. In contrast, the transcripts and proteins of KSHV latent genes like LANA, vFLIP and vCyclin were unchanged between group1 and group2 or between group3 and group4 (S2C–S2F Fig), indicating Notch activation had no effect on the expression of KSHV latent genes. Additionally, we also sorted co-cultured SLK-RTA and SLK-Ctrl cells and checked RTA and JAG1 expression as well. Consistently, there is no difference between co-cultured system and separately cultured system as JAG1 was still highly expressed in SLK-RTA cells (S2I–S2L Fig compared with S2A Fig). Taken together, these results suggested that cells with high level of JAG1 expression induced by RTA can activate the Notch signaling pathway in neighboring cells and negatively control viral lytic reactivation in those cells.

### Notch inhibition promotes KSHV reactivation in a pro-lytic environment

Based on above observations, we inferred that inhibitors of the Notch pathway might potentiate KSHV reactivation and facilitate lytic reactivation. To validate this hypothesis, the γ-secretase inhibitor DAPT was used to block Notch pathway in iSLK.RGB cells with doxycycline stimulation or in the context of hypoxia to mimic physiological stresses. To avoid the artifacts of DAPT itself on KSHV transcription profiles, iSLK.RGB cells were first treated with either DMSO or DAPT for 12, 24 and 48 h, and cells were harvested for viral RNA analysis at each time point. The selected lytic gene transcripts were not up-regulated (S3A Fig). Moreover, the expression of doxycycline induced RTA in iSLK cells was not influenced by DAPT treatment (S3B Fig), which get rid of the concern that DAPT would affect doxycycline induction system and subsequent lytic gene expression in iSLK.RGB cells.

**Fig 3:**
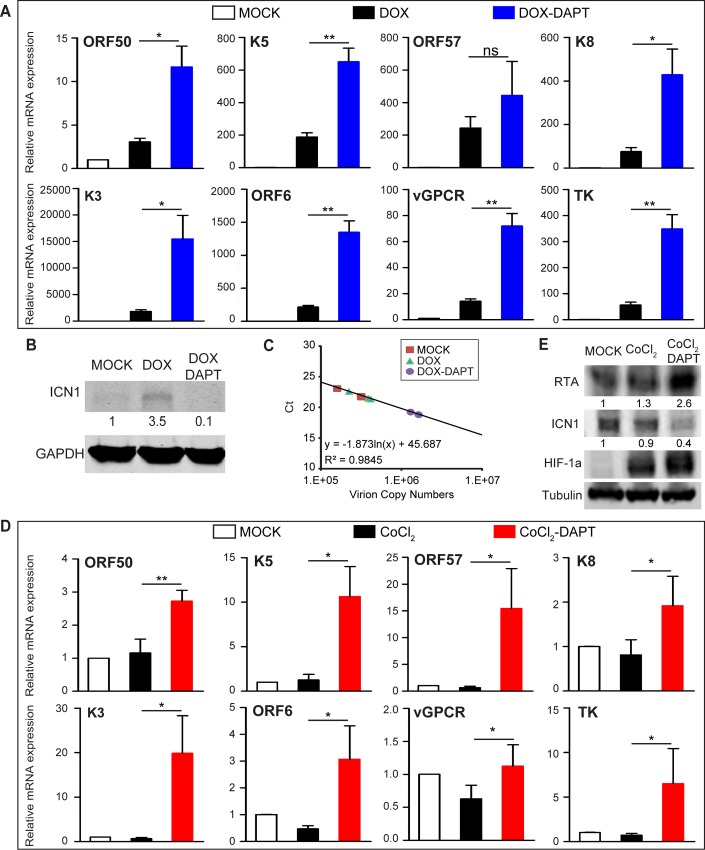
Notch pathway inhibition up-regulates lytic gene expression. (A) iSLK.RGB cells were plated in 6-well plates at 0.1 million cells per well. Quantification of the transcripts of the indicated KSHV lytic genes by qPCR after DMSO, doxycycline or doxycycline plus DAPT (40 μM) treatment for 36 h in DMSO or DAPT pre-treated iSLK.RGB cells for 12 h. (B) Western blotting analysis of ICN1 levels in extracts from iSLK.RGB cells treated with DMSO, doxycycline or doxycycline plus DAPT. (C) Quantification of relative KSHV genome copy number from the culture supernatant by qPCR after DMSO, doxycycline or doxycycline plus DAPT (40 μM) treatment for 36 h in DMSO or DAPT pre-treated iSLK.RGB cells for 12 h. (D) The transcripts of the indicated lytic genes were quantified in iSLK.RGB cells treated with DMSO, CoCl_2_ (200 μM) or CoCl_2_ (200 μM) plus DAPT (40 μM) for 36 h in 6-well plates. (E) RTA, ICN1 and HIF-1α expressions were detected by western blotting from whole cell extracts treated as in (D). Data were expressed as the mean ± s.e.m., n = 3, *p<0.05, **p<0.01, ***p<0.001.

To explore the effect of DAPT on KSHV lytic reactivation and viral particle production in the presence of RTA induced by doxycycline, iSLK.RGB cells were pretreated with DAPT for 12 h and then stimulated with doxycycline for another 36 h before analysis. With DAPT completely abrogating RTA-induced ICN1 production (Fig 3B), a significantly higher lytic gene induction was observed in response to doxycycline stimulation compared with that in cells without DAPT treatment (Fig 3A). As a result, KSHV viral progenies were produced at a higher level in DAPT treated cells than those in the DMSO group (Fig 3C). The same results were obtained by using another Notch inhibitor, LY-411575 (S3C–S3E Fig).

Previous studies showed that hypoxia reactivates KSHV from latency [11–13]. The key hypoxia responder hypoxia-inducible factor 1α (HIF-1α), which binds and activates the RTA promoter, is stabilized by KSHV [11–14]. KS develops in a setting of relatively low oxygen, and such a hypoxic environment may play a pivotal role in KSHV spontaneous reactivation, which facilitates virus spread and re-infection. To examine whether Notch regulates viral reactivation in the context of hypoxia, cobalt chloride (CoCl_2_), a chemical inducer of hypoxia, was used to test the effect of DAPT treatment on KSHV lytic induction. Few KSHV lytic genes responded to CoCl_2_ treatment (Fig 3D) despite the induction of HIF-1α (Fig 3E). The relative unresponsiveness to hypoxia in iSLK.RGB might be the reason that iSLK.RGB tightly controlled its reactivation, the mechanism of which is not completely known [37]. However, blocking Notch signaling with DAPT (Fig 3E) resulted in a dramatic up-regulation of lytic gene expression at both mRNA and protein levels (Fig 3D and 3E) in the presence of CoCl_2_. These results reinforced the hypothesis that the Notch pathway acted as a negative regulator of lytic genes during KSHV reactivation.

### JAG1 and Notch1 negatively regulate RTA-induced KSHV lytic replication

**Fig 4:**
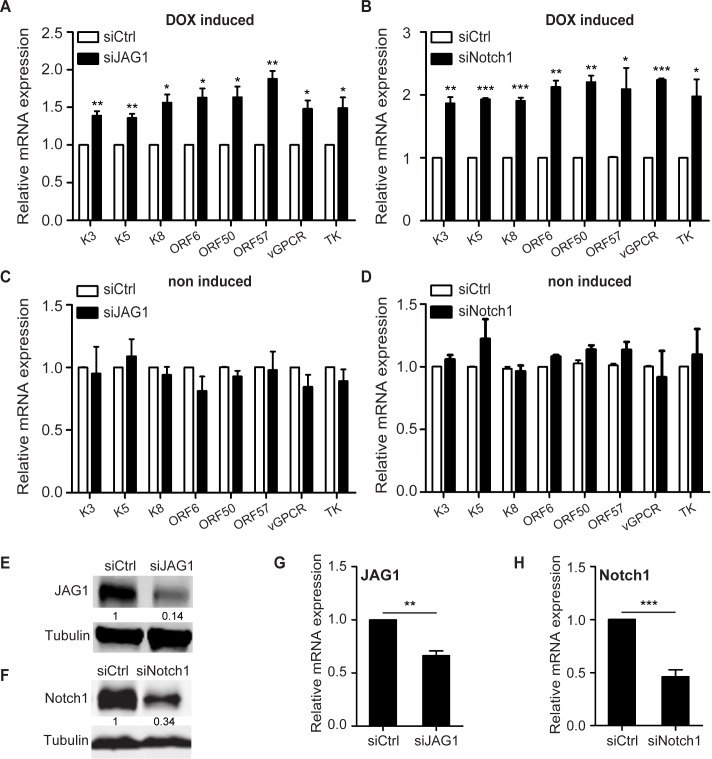
Knockdown of JAG1 or Notch1 up-regulates lytic gene expression. (A, C) Quantification of the expression of the indicated lytic genes in iSLK.RGB cells transfected with siRNA (100 pM per well) against JAG1 (siJAG1) or scramble controls (siCtrl) in 6-well plates for 8 h when cells reached 60% confluence and treated with (A) or without doxycycline (C) for another 24 h. (B, D) Quantification of the expression of the indicated lytic genes in iSLK.RGB cells transfected with siRNA (100 pM per well) against Notch1 (siNotch1) or scramble controls (siCtrl) and treated with (B) or without doxycycline (D). Values represent the mean ± s.e.m., n = 3, *p<0.05, **p<0.01, ***p<0.001. (E–H) The efficiency of JAG1 or Notch1 depletion was detected by western blotting (E, F) and qPCR (G, H).

Although DAPT shows inhibitory activity on Notch signaling, it also suppresses the proteolysis of several other transmembrane proteins, including LDL receptor-related protein [38], E-cadherin [39], and Receptor tyrosine-protein kinase erbB-4 [40]. To examine whether Notch signaling specifically contributed to the inhibition of KSHV reactivation, Notch pathway components were silenced by siRNA with high efficiency in iSLK.RGB cells treated with doxycycline. At 8 h after JAG1 siRNA transfection, cells were induced for reactivation for another 36 h and subjected to RNA extraction. JAG1 was significantly down-regulated at the mRNA and protein levels (Fig 4E and 4G), and this was associated with the up-regulation of lytic genes compared with the levels in the siCtrl group in response to RTA induction (Fig 4A).

As JAG1 binds to Notch receptors and Notch1 was up-regulated in response to reactivation, as shown in Fig 1 (Fig 1A and 1C), we speculated that knockdown of Notch1 would reproduce this change in the expression of KSHV lytic genes. iSLK.RGB cells were transfected with siRNA against Notch1, and lytic gene expression was quantified. The induction of lytic genes was increased (Fig 4B) in line with the down-regulation of Notch1 (Fig 4F and 4H). The effects of JAG1 or Notch1 depletion were confined to the lytic phase, as no difference in gene expression was observed in the absence of doxycycline treatment between siJAG1/siNotch1 group and the siCtrl group (Fig 4C and 4D). These results indicated that after the initiation of lytic reactivation, the Notch signaling pathway activated by RTA plays a role in preventing viruses from undergoing explosive lytic replication.

### Hes1 inhibits the transactivation of KSHV lytic genes

**Fig 5:**
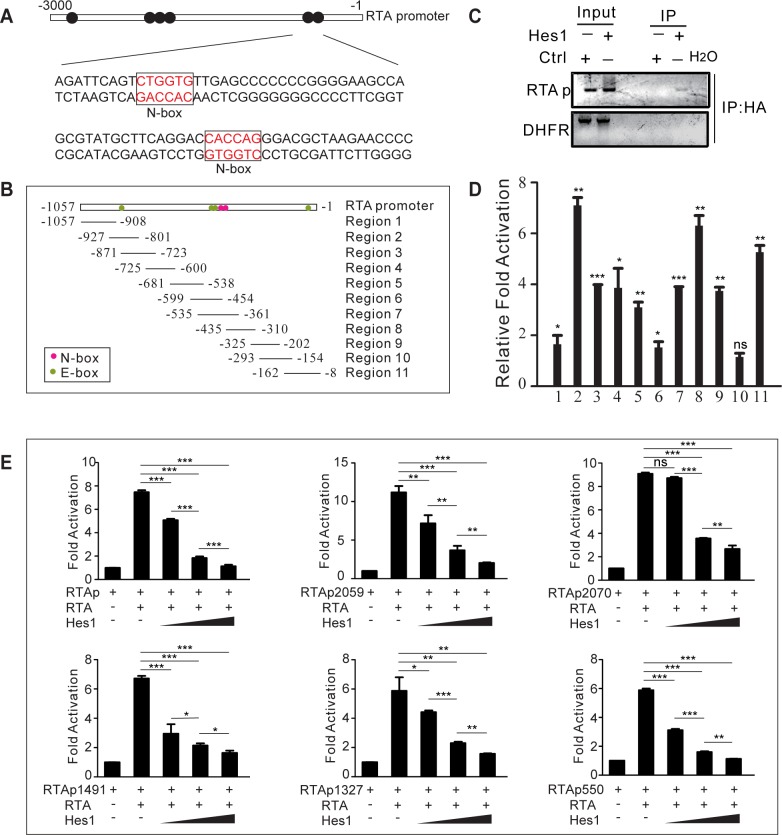
Hes1 inhibits RTA self-transactivation. (A) Schematic illustrating the N-box Hes1 binding motifs within the RTA promoter. (B) Primers were designed to cover the RTA promoter. (C, D) 293.219 cells plated in 100 mm dish at 4 million cells 24 h before transfection. HA-Hes1 and control plasmids (12 μg) were transfected and ChIP assay was performed 24 h after transfection. Extracts were subjected to immunoprecipitation with an anti-HA-tag antibody and purified DNA elute was quantified by gel analysis (C) or qPCR with the indicated primers from B (D). (E) Hes1 represses the transcriptional activity of the RTA promoter. Dual luciferase assay was performed in HEK293T cells plated in 12-well plates at 0.5 million per well 24 h after transfection. The cells were transiently transfected with expression vectors containing RTA (1 μg), reporter plasmids containing full length RTA promoter or RTA promoter truncations (100 ng), and increasing amounts of Hes1 (250 ng, 500 ng or 1μg) using Lipofectamine 2000. Total transfected DNA was normalized with pcDNA3.1. Data were expressed as the mean ± s.e.m., n = 3, *p<0.05, **p<0.01, ***p<0.001.

We next explored the mechanism by which activated Notch inhibits KSHV reactivation. Hes1 functions as a downstream effector and a feedback inhibitor of the Notch pathway and suppresses various genes expression by directly binding with DNA [41]; Hes1 negatively modulates gene expression by binding to consensus DNA sequences, defined as N-box (CACNAG) with high affinity and E-box (CANNTG) with low affinity [41]. Inspection of the promoter regions of a number of KSHV lytic genes revealed several conserved N-box and E-box motifs (S1 Table). Multiple N-box and E-box motifs were found in the RTA promoter (Fig 5A and 5B), indicating its strong potential to be regulated by Hes1. Therefore, we speculated that the up-regulated Hes1 in response to Notch activation would in turn suppress the expression of KSHV lytic genes.

To determine whether Hes1 is recruited to the RTA promoter region, ChIP assay was performed against Hes1 in 293.219 cells (HEK293T cells stably infected with recombinant KSHV virus 219) with ectopic HA-Hes1 expression. The results showed that Hes1 bound specifically to the RTA promoter region containing the Hes1-binding sites, as evidenced by the specific DNA band amplified in the Hes1 transfection group but not in the control group (Fig 5C). To verify the binding sites of Hes1 on the RTA promoter, 11 pairs of primers encompassing over 1000 base pairs of the RTA promoter (from -1 to -1057), which potentially contains four E-box and two N-box motifs, were designed (Fig 5B). Hes1 occupied the E-box or N-box regions (regions 2, 8, and 11) with high affinity (Fig 5D). Notably, we observed a comprehensive binding capability of Hes1 throughout the RTA promoter, and this was probably due to its interaction with other regulatory factors, such as Sirtuin 1 [42], which binds to the RTA promoter and negatively regulates KSHV lytic replication [43].

As a suppressive bHLH molecule that represses RBP-Jκ target gene expression [41], Hes1 may also antagonize RTA-mediated lytic gene transactivation. Therefore, the effect of Hes1 on the ability of RTA to activate its own promoter was tested. A dual luciferase assay was performed with HEK293T cells transiently transfected with RTA expression plasmids, reporter plasmids containing full-length RTA promoters or various RTA promoter truncations, and increasing amounts of Hes1. RTA promoted the activation of the reporters, whereas co-expression of Hes1 inhibited the transactivation activity of RTA on its own promoter in a dose-dependent manner (Fig 5E). To determine whether Hes1 affected the epigenetic modification of RTA promoter and repressed RTA expression. We performed ChIP assay to evaluate the activating histone H3 Acetylation (H3Ac) and activating H3K4 trimethylation (H3K4me3) at RTA promoter. Both H3Ac and H3K4me3 were reduced throughout RTA promoter in the presence of Hes1, indicating a lower promoter activity (S4A and S4B Fig).

**Fig 6:**
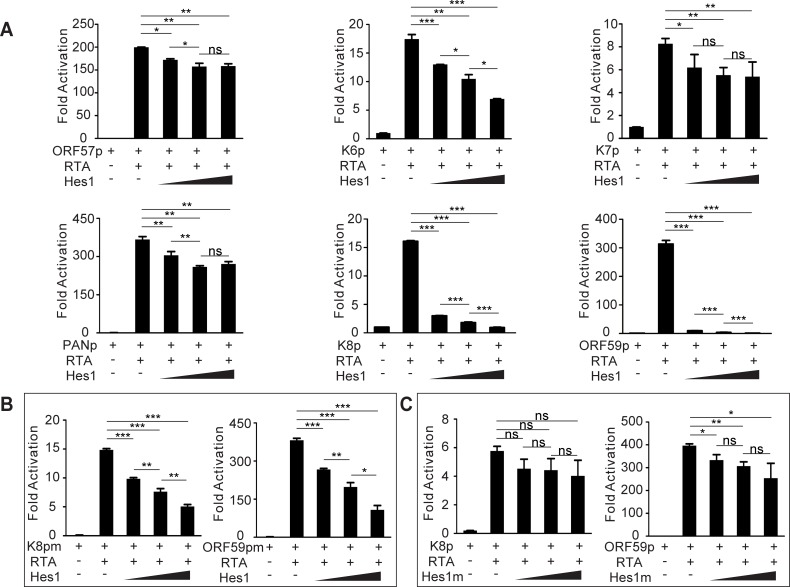
Hes1 inhibits RTA-induced activation of KSHV lytic genes. (A) HEK293T cells were plated into 12-well plates at 0.5 million cells per well, a dual luciferase assay was performed in HEK293T cells transiently transfected with expression vectors containing RTA (1 μg), various reporter plasmids containing lytic gene promoters (100 ng), and increasing amounts of Hes1 (250 ng, 500ng or 1 μg) using Lipofectamine 2000. Total transfected DNA was normalized with pcDNA3.1. (B, C) A dual luciferase assay was performed in HEK293T cells transiently transfected with different vector combinations. (B) Mutant K8 (Left) or mutant ORF59 (Right) promoters were transfected with RTA and increasing amounts of Hes1. (C) Mutant Hes1 or wild type Hes1 containing plasmids were transfected with RTA and K8 or ORF59 promoters. Data were expressed as the mean ± s.e.m., n = 3, *p<0.05, **p<0.01, ***p<0.001.

Next we assessed the effects of Hes1 on other lytic genes transactivated by RTA. ChIP assay showed that Hes1 was enriched around Hes1 binding sites (N-box and E-box) in the lytic gene promoters respectively (S4C and S4D Fig). As expected, Hes1 inhibited the activation of lytic promoters induced by RTA in a manner dependent on the number and nature of Hes1 binding sites in the promoter regions (Fig 6A). In genes such as K8 and ORF59, which are highly susceptible to Hes1 inhibition, deletion of the N-box motifs in the corresponding promoters strongly counteracted the repressive activity of Hes1 (Fig 6B). Consistent with the promoter mutation results, co-expression of RTA with mutant Hes1 which is deficient in interacting with Grouch/TLE co-repressors reversed Hes1-induced transactivation inhibition (Fig 6C). It was worth to be noted that Hes1 antagonized lytic gene promoter activity was independent of RTA as Hes1 still inhibited the activation of RTA and other lytic promoters without RTA expression (S5 Fig).

In order to further understand the significance of Hes1 in suppressing KSHV lytic gene expression, we reduced down the Hes1 levels by DAPT and performed co-culture assays to see the effects of Notch/Hes1 axis during KSHV reactivation. Three groups (group5, 6, and 7) of RFP and GFP double positive iSLK.RGB cells were sorted from three co-culture mixtures respectively: (1) iSLK.RGB treated with DMSO and co-cultured with SLK-Ctrl cells in the presence of doxycycline; (2) iSLK.RGB treated with DMSO and co-cultured with SLK-RTA cells in the presence of doxycycline; (3) iSLK.RGB treated with DAPT and co-cultured with SLK-RTA cells in the presence of doxycycline (S6A Fig). In agreement with Fig 2F, the expression of lytic genes was lower in group6 compared with that in group5 (S6B Fig). However, when treated with DAPT to reduce down the Hes1 expression (S6C Fig), the expression of lytic genes was restored and even higher in group7 compared with that in group5 (S6B Fig).

Taken together, these results indicated that Hes1, as a downstream effector of Notch signaling, was critical for regulating a broad spectrum of KSHV lytic genes by directly binding to lytic gene promoters and controlling the equilibrium between latency and reactivation.

### RTA modulates JAG1 expression transcriptionally by binding to LEF1 and interfering with the LEF1/TLE transcription suppressive complex

**Fig 7:**
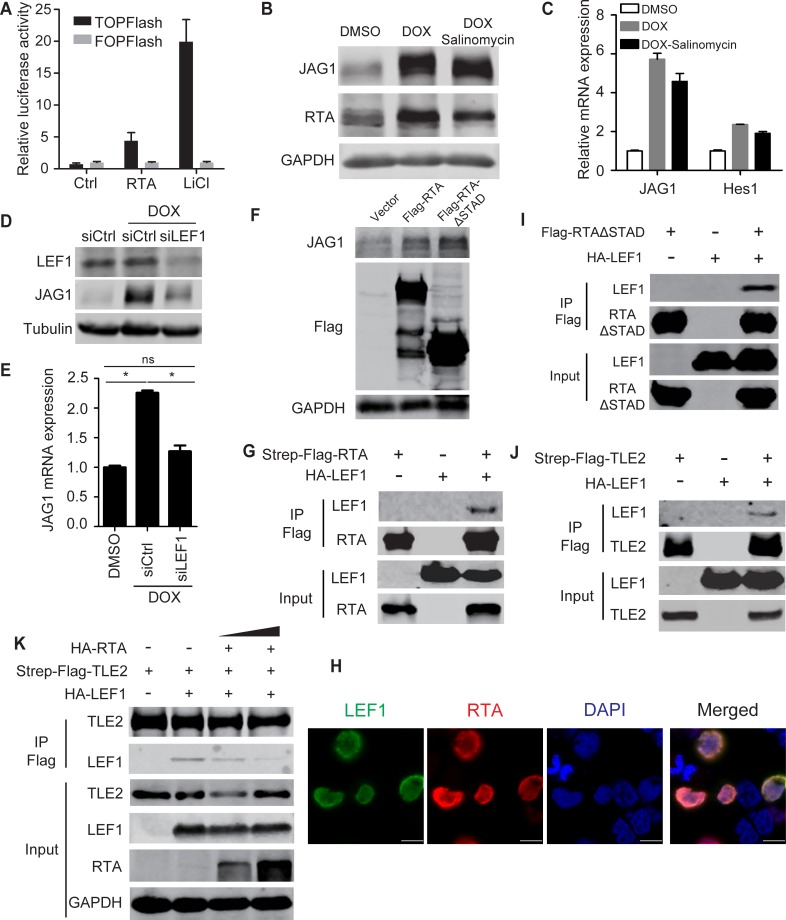
RTA interacts with transcription factors downstream of the Wnt pathway to up-regulate JAG1. (A) A dual luciferase assay was performed in HEK293T cells plated in 12-well plates co-transfected with RTA or vector plasmids (2 μg each) and Wnt reporter plasmids TOP/FOPFlash (0.2 μg). LiCl (20mM) treatment was used as positive control. (B, C) Quantification of JAG1 expression by western blotting and qPCR in iSLK cells (70–80% confluence) treated with doxycycline (5 μg/ml) plus DMSO or salinomycin for (2μM) 24 h in 6-well plates. (D, E) JAG1 expression was quantified in iSLK cells transfected with siRNA (200 pM) against LEF1 (siLEF1) or scramble control (siCtrl) for 8 h when cells reached 60% confluence and treated with doxycycline for another 24 h. (F) JAG1 protein levels were quantified by western blotting in HEK293T cells transfected with RTA-SF, RTA-ΔSTAD or vector plasmids (4 μg each). (G, I, J) Co-immunoprecipitation assays were performed in HEK293T cells plated in 100 mm dish to test the physical interactions between SF-RTA and HA-LEF1 (12 μg each) (G), SF-RTA-ΔSTAD and HA-LEF1 (12 μg each) (I), and SF-TLE2 and HA-LEF1 (12 μg each) (J). (H) Immunofluorescence assay was performed in HEK293T cells transfected with SF-RTA and HA-LEF1 plasmids. Green color indicates LEF1 expression, Red color indicates RTA expression. Scale bars represent 10 μm. (K) A competition co-immunoprecipitation assay was performed in HEK293T cells plated in 100 mm dish to test RTA disrupted the LEF1/TLE2 complex. SF-TLE2 (4 μg) was co-transfected with HA-LEF1 (4 μg) alone or co-transfected with HA-LEF1 (4 μg) and increasing amounts of HA-RTA (4 and 8 μg). Precipitated HA-LEF1 was analyzed by western blotting.

Lastly, we explored the mechanism by which RTA up-regulates JAG1 transcriptionally. JAG1 is induced by several intracellular signaling pathways, including NF-κB [44], Notch [35, 36], and Wnt pathway [45, 46]. However, the expression and phosphorylation of the NF-κB core components remained unchanged in doxycycline treated iSLK cells compared with untreated counterparts (S7A Fig). Furthermore, the NF-κB inhibitor BAY11-7082 failed to block the induction of JAG1 and the downstream effectors Hes1 and Hey1 induced by RTA (S7B and S7C Fig). Similarly, Notch inhibitor DAPT did not reverse the up-regulation of JAG1 in doxycycline treated iSLK cells (S7D and S7E Fig). Since the NF-κB and Notch pathways were not involved in the RTA-induced up-regulation of JAG1, we investigated whether RTA can activate JAG1 expression through Wnt signaling pathway. Treatment of doxycycline-induced iSLK cells with the Wnt inhibitor salinomycin [47] for 16 h had moderate effect on JAG1 expression at the mRNA and protein levels (Fig 7B and 7C), suggesting that JAG1 was induced by RTA in a manner independent of Wnt/β-catenin signaling. However, RTA alone significantly induced TOPFlash activity and had no effect on FOPFlash (Fig 7A). As the TOPFlash reporter contains multimeric LEF1 (a transcription factor that interacts with β-catenin [48]) binding sites and FOPFlash contains mutant LEF1 binding sites, we speculated that LEF1 may act as a key factor in RTA-Wnt pathway crosstalk. To test this hypothesis, LEF1 expression was silenced by siRNA and RTA was induced by doxycycline. The level of JAG1 was reduced to the baseline in the siLEF1 group (Fig 7D and 7E), suggesting that RTA regulates JAG1 through LEF1. Furthermore, the transactivation activity of RTA was not required for JAG1 up-regulation, as overexpression of truncated RTA lacking the transactivation domain (RTA-ΔSTAD) enhanced JAG1 expression to the same level as full-length RTA (Fig 7F). This indicated that RTA may up-regulate JAG1 expression by interacting with LEF1. We then performed co-immunoprecipitation (Co-IP) experiments to confirm that RTA physically interacted with LEF1 in HEK293T cells (Fig 7G). In addition, immunofluorescence assays showed that RTA co-localized with LEF1 in the nucleus (Fig 7H). The Co-IP assay also proved the interaction between RTA-ΔSTAD and LEF1 (Fig 7I). In the absence of an activating signal, the LEF1 protein serves as a transcription repressor by directly binding with the Q domain of Groucho/TLE, and this interaction can be disrupted by β-catenin upon Wnt activation [49–51]. In this context, we also confirmed that LEF1 can interact with transducin-like enhancer protein 2 (TLE2) by Co-IP (Fig 7J). In our previous study, we showed that TLE2 functions as a RTA binding protein and represses RTA auto-activation and transactivation activities [52]. Interestingly, RTA also interacted with the Q domain of TLE2, which is shared by LEF1. Based on these findings, we explored whether RTA was able to disrupt the interaction between TLE2 and LEF1. HEK293T cells were transfected with SF-TLE2 with or without HA-LEF1 plasmids along with different amounts of RTA. Increasing amounts of RTA co-expressed in the cells resulted in the competitive exclusion of LEF1 from the TLE2/LEF1 complexes in a dose-dependent manner (Fig 7K). These data suggested that RTA can release LEF1 from the TLE/LEF1 inhibitory complex and up-regulate JAG1 expression.

## Discussion

KSHV mainly displays latency in spindle cells; however, in a small subset of infected cells, viruses spontaneously switch into the lytic phase. The latency program plays a primary role in KSHV associated tumorigenesis, whereas lytic replication allows the virus to spread throughout the host and induces an inflammatory environment during pathogenesis [19, 53].

Many factors contribute to form a pro-lytic microenvironment during disease progression. To our knowledge, all KS lesions contain significant numbers of cells like B cells, plasma cells, CD4+ T cells, CD8+ T cells, monocytes, and high levels of IFN-gamma [19, 54], which forms a tumor microenvironment likely to induce KSHV reactivation. Aside from the inflammatory conditions, hypoxia is another common factor that arises in tumor, which reactivates KSHV from latency [11–14, 55]. Clinical observation also proved that KS tumors often occur on body parts with low oxygen supply such as feet, mouth and arms [19]. Reactive oxygen species (ROS) is another KSHV reactivation inducer [15, 16]. The higher amount of ROS is a common factor generated by tumor metabolic alteration [56]. Similar with other tumors, KS displays Warburg-like effect which leads global metabolic changes [57–60] and stresses the cells via ROS production, thereby reactivates KSHV from latency. KS is a common cancer in AIDS patients and organ transplant patients with immunosuppression. HIV-1 infection has been demonstrated to trigger KSHV reactivation in PEL cells, probably by activating RTA promoter [61, 62]. Moreover, HIV-1 Tat protein might be an important factor potentially to initiate lytic cycle of KSHV [63]. With immunosuppression, a lot of other pathogens took this opportunity to infect patients. Studies showed that super-infection with other pathogens like HSV-1, HHV-6, cytomegalovirus (CMV) causes KSHV reactivation [64–66].

These factors are common in KS lesions, although reactivation is a rare event in tissue with 1 to 3% of spindle cells displaying lytic replicative markers [6–9, 19, 67]. This indicates the presence of specific intrinsic regulatory strategies that allow KSHV to fine-tune the balance between latency and reactivation in virus infected cells.

**Fig 8:**
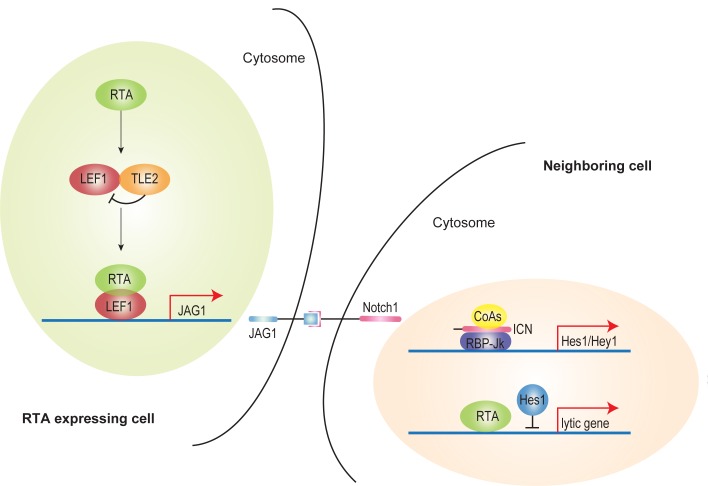
A proposed working model. RTA up-regulates JAG1 expression by disrupting LEF1/TLE suppressive complex via competitively binding with LEF1. Elevated membrane-bound JAG1 in RTA expressing cells interacts with Notch receptors and subsequently activates Notch signaling by producing higher level of Notch Intracellular Domain (ICN) in neighboring cells. Notch downstream effector Hes1 is up-regulated in response to activated Notch signaling. Hes1 directly binds and suppresses RTA and lytic gene promoters which in turn inhibits KSHV reactivation in the context of a specific microenvironment that drives lytic replication.

Our study provides a novel regulation mode for this phenomenon (Fig 8). Previously, we showed TLE2 was a novel RTA binding protein which independently inhibited the ability of RTA to auto-activate its own promoter [52]. Grouch/TLE co-repressors did not bind DNA directly; instead, they recruited a diverse profile of transcription factors, including Hes, LEF1/TCF and c-Myc etc. to elicit its functions [68]. The repressive complex formed by Grouch/TLE and TCF/LEF could be broken down by β-catenin induced by Wnt activation [46]. JAG1 promoter contains several LEF1 binding motifs and can be activated via interaction with LEF1 [46]. As RTA dramatically up-regulated JAG1 expression (Fig 1A–1D) and RTA co-precipitated with LEF1 (Fig 7G), RTA therefore released LEF1 from TLE/LEF1 suppressive complex, which subsequently recruited other co-activators to drive JAG1 expression (Fig 7D, 7E and 7K). Membrane-bound JAG1 activated Notch signaling *in trans* by binding with Notch receptors in neighboring cells (Fig 2B–2D). Hes1, as downstream effectors of Notch signaling, was a potent transcriptional repressor which bound directly with DNA. Hes1 binding motifs could be found in the promoters of various lytic gene promoters including RTA. Thus Hes1 induced by activated Notch broadly suppressed KSHV lytic reactivation in neighboring cells signaled by RTA expressing cells (Fig 8). This provides a possible explanation for the fact that lytic replication of KSHV occurs only in a small portion of cells within a relatively consistent tumor microenvironment.

Aberrant Notch activation was previously reported in KSHV-infected cells and KS tumor samples [20, 69–74]. The Notch ligands JAG1 and Dll4 and the Notch receptors Notch1–4 are functionally overexpressed in KS tumor cells [20]. KSHV-encoded viral FLICE-inhibitory protein (vFLIP) induces JAG1 expression in NF-κB dependent manner, and vGPCR up-regulates Dll4 by activating ERK in LEC cells [69]. Other KSHV ORFs (LANA, RTA, and v-IL6) also regulate Notch components [70]. The activated Notch plays a pivotal role in KSHV induced pathogenesis. In LEC, KSHV-induced Notch activation down-regulates prospero homeobox protein 1 (PROX1), a master regulator of lymphatic development, through Hey1 and drives LECs to reprogram into BECs [71]. JAG1 and Dll4 up-regulated by vFLIP and vGPCR induce membrane-type-1 matrix metalloproteinase (MT1-MMP)-dependent endothelial-to-mesenchymal transition (EndMT), facilitating the invasion of surrounding connective tissues by the infected cells [72]. The up-regulated JAG1 and Dll4 also lead to cellular quiescence in neighboring cells [69, 72]. In previous work, we showed that LANA stabilizes intracellular activated Notch (ICN) by competing with ICN for binding with Sel10 (a negative regulator of Notch) and maintains the enhanced proliferation and angiogenesis of KSHV-infected cells [73, 74].

In addition to its involvement in the pathogenesis of diseases, Notch signaling plays a critical role in asymmetric cell division and fate determination, which is reflected by distinct gene expression among cells. In signal receiving cells, Notch is activated by the binding of Notch receptors to membrane-anchored Notch ligands on neighboring cells (signal donor cells) where Notch signaling is inhibited by the ligand intracellular domain and other regulatory factors [27, 75], which is known as lateral inhibition. Our results suggest that asymmetric gene expression induced by lateral inhibition affects the behavior of KSHV in infected cells. Notch distinguishes the lytic phase of KSHV in “RTA already expressing” cells and “RTA ready to express” cells, as evidenced by the co-culture assay showing that cells stably expressing RTA inhibit lytic gene activation in cells induced to express RTA at a later time. KSHV usurps this pathway to strategically control the amounts of free-floating viruses and the apoptosis of infected cells, thus evading the host defense mechanisms. The results of the present study suggest a novel role of Notch signaling in virus fate determination.

Our results showed the cell-to-cell connection in the monolayer 2D adherent cell culture. In the paper published by Ojala group [72], Cheng and coauthors established a 3D culturing system that better mimics cell-to-cell interactions occurring *in vivo*. KSHV infection in 3D environment induced a dramatic outgrowth of capillary sprouts and mesenchymal phenotype transition (EndMT). The EndMT sprouting process was Notch dependent as Notch inhibitors suppressed sprouting of KSHV infected LEC (K-LEC) spheroids. Interestingly, the majority of K-LEC spheroids displayed higher numbers of nuclear punctate LANA signal and increased viral DNA levels of the cells while lytic genes could also be detected in the cells, in agreement with KSHV infection *in vivo*. Thus, in 3D environment or *in vivo* conditions, activated Notch not only facilitates the interaction of cells but also improves maintenance of viral episomes, probably achieved by *in trans* reactivation inhibition.

In summary, Notch is implicated in KSHV-associated malignancy and contributes to KS development in multiple phases. On one hand, activated Notch promotes key events in tumor progression, such as sustained proliferation, angiogenesis, and EndMT-mediated cell invasion [72–74]; on the other hand, the fine-tuned Notch signaling stringently controls viral reactivation, enabling long-term persistence of KSHV in host cells. Our work not only provides a molecular mechanism to explain how the balance between viral lytic replication and latency is maintained in infected cells, but also suggests that this fine-tuned regulation is an important evolutionary strategy utilized by KSHV to survive in the host.

## Materials and Methods

### Cell lines and plasmids

The TRE-BCBL1-RTA cell line was maintained in RPMI 1640 medium (Hyclone) containing 10% fetal bovine serum (FBS) and 1% antibiotics (penicillin and streptomycin, Hyclone). The HEK293T, 293.219, iSLK and iSLK.RGB cell lines were cultured in DMEM (Hyclone) supplemented with 10% FBS (Hyclone) and 1% antibiotics (penicillin and streptomycin, Hyclone). We are grateful to Dr. Jae Jung (University of Southern California) and Dr. Fanxiu Zhu (The Florida State University) for the TRE-BCBL1-RTA, iSLK.RGB, and iSLK cell lines; Dr. Don Ganem (Novartis Institutes for Biomedical Research) for the 293.219 cell line. The HEK293T cell line was from our laboratory stock.

A modified pCDH-SF-IRES-Blast vector was constructed by replacing EF1 with internal ribosome entry site (IRES), and introducing a fragment encoding tandem Strep-tag II and Flag peptide. The selection marker puromycin was replaced by blasticidin. pCDH-SF-RTA-IRES-Blast or pCDH-SF-RTA-ΔSTAD-IRES-Blast was generated by cloning full-length RTA or RTA-ΔSTAD into the modified vector. pCMV-HA-LEF1 and pCMV-HA-Hes1 was constructed by amplifying LEF1 and Hes1 from HEK293T cDNA and inserting the PCR products into the pCMV-HA vector. The Hes1 mutant plasmid was constructed by site-directed mutagenesis following the manufacturer’s protocol (Quick Change Mutagenesis Kit; Stratagene). The SF-tagged TLE2 expression plasmid pCDH-SF-TLE2, the luciferase reporter plasmids pGL3-PANpluc, pGL3-ORF57pluc, pGL3-ORF59pluc, pGL3-K8luc, pGL3-RTApluc, and a series of truncation plasmids of pGL3-RTApluc were described previously [32, 52]. The reporter plasmids pGL3-K6pluc and pGL3-K7pluc contained the PCR-cloned promoter regions of ORFK6 (nt 27577 to 28576) and ORFK7 (nt 27773 to 28772), respectively. The promoter fragments were inserted into the NheI/HindIII sites upstream of the luciferase reporter gene of the pGL3-basic vector. pGL3-ORF59p-mutant-luc was constructed by amplifying ORF59 promoter sequence from pGL3-ORF59pluc using ORF59-mutant primers listed in S2 Table. pGL3-K8p-mutant-luc was obtained by performing a one-step mutation PCR on pGL3-K8pluc, and then subcloned into the pGL3-basic vector. The Top/FopFlash plasmids were kindly provided by Dr. Lin Li (Institute of Biochemistry and Cell Biology, SIBS, CAS). All primers for gene amplification and qPCR are listed in S2 Table.

The siRNA sequences used in the study are as follows:

siCtrl: 5′- AAUUCUCCGAACGUGUCACGU-3′;
siNotch1: 5′- AAGGUGUCUUCCAGAUCCUGA-3′;
siJAG1: 5′-CCGGATGGAATACATCGTATA-3′;
siLEF1: 5′-CCAUCAGAUGUCAACUCCAAA-3′.

### Antibodies and reagents

The following primary antibodies were used: anti-RTA mouse monoclonal antibody was prepared in our laboratory; anti-Notch1 (Cell Signaling technology, #4380P), anti-cleaved Notch1 (Cell Signaling technology, #4147P), anti-JAG1 (Cell Signaling technology, 2620P), anti-HIF1-α (BD Biosciences, 610958), anti-acetyl-Histone H3 (Merck Millipore, 06–599), anti-H3K4me3 (Abcam, ab8580), anti-p-IKKα/β, anti-IKKα, anti-IKKβ, anti-IκBα, anti-p-IκBα, anti-p65 (Cell Signaling technology, 9958), anti-Flag (Sigma, F7425 and F1804), and anti-HA (Sigma, H9658 and H6908). The secondary antibodies were as follows: goat anti-mouse IRDye@800cw (LI-COR, 926–32210), goat anti-rabbit IRDye@800cw (LI-COR, 926–32211), goat anti-rabbit IgG(H+L) Alexa Fluor@488 (Invitrogen, R37116), and goat anti-mouse IgG(H+L) Alexa Fluor@555 (Invitrogen, A-21422). Other reagents used and their sources were as follows: anti-Flag M2 affinity gel (Sigma, A2220), DAPT (Sigma, D5942), LY-411575 (Sigma, SML0506), NF-κB inhibitor BAY 11–7082 (Beyotime, S1523), Wnt inhibitor Salinomycin (Sigma, S4526), and TNFα (Peprotech, 315-01A).

### Quantitative real-time PCR (qPCR)

Cells were harvested and lysed with the TRIzol reagent (Life Technologies). Total RNA was extracted according to the manufacturer’s instructions. Two micrograms of total RNA were used to reverse transcribe cDNA with the genomic DNA eraser RT kit (TaKaRa). Quantitative real-time PCR (qPCR) was performed with a SYBR green Master Mix Kit (Toyobo). Primers are listed in S2 Table. Relative mRNA levels were normalized to GAPDH and calculated by the ΔΔCt method. Fold activation levels were calculated against control samples. Samples were tested in triplicate. The quantification of extra-cellular virion copy number was described previously [52].

### Co-culture assay and flow cytometry-assisted cell sorting

The cell lines SLK-RTA and SLK-Ctrl were constructed by lentiviral transduction and blasticidin selection. SLK-RTA and SLK-Ctrl cells were co-cultured with iSLK.RGB cells at a ratio of 1:1 for 24 and 48 h. RFP positive iSLK.RGB cells were sorted by FACS and subjected to qPCR analysis or western blotting. For the detection of lytic gene expression, SLK-RTA and SLK-Ctrl cells were co-cultured with iSLK.RGB cells at a ratio of 1:1 for 24 h and then doxycycline was added to induce RTA expression. At 36 h post-induction, the iSLK.RGB cells that were RFP and GFP double positive were sorted by FACS and subjected to qPCR analysis or western blotting.

### Dual-luciferase reporter assay

A dual-luciferase reporter assay system (Promega) was used according to the manufacturer’s protocol. The pRL-SV-40 plasmids expressing *Renilla* luciferase activity were used to normalize firefly luciferase activity. The luciferase plasmid together with plasmids expressing RTA or Hes1 were transfected into HEK293T cells seeded in 12-well plate, and the total amount of DNA was normalized to that of the empty vector in the transfection. At 24 h post-transfection, cells were harvested to detect luciferase activity. The results are expressed as the fold change relative to cells transfected with empty vectors.

### Chromatin immunoprecipitation assay (ChIP)

The ChIP assay was performed as previously described. Briefly, protein A/G beads (Invitrogen) were precleared with binding buffer containing 0.2 mg of salmon sperm DNA per ml for 6 h. The 293.219 cells were transfected with HA-Hes1 or control vector. At 24 h after transfection, cells were crosslinked with formaldehyde at 1% final concentration for 15 min. Glycine was added to quench the formaldehyde with a final concentration of 125 mM. Cells were lysed with SDS lysis buffer containing a protease inhibitor (PMSF). The lysate was sonicated to shear the chromatin to an average length of 750 bp, and 5 μg of indicated antibodies were added to the lysates and incubated at 4°C overnight. The precleared beads were added into each sample at 4°C for 2 h for immunoprecipitation. To extract the DNA fragments, TE buffer with 1% SDS and proteinase K (Beyotime) was added to the washed precipitates. After incubation at 65°C for at least 6 h for reverse crosslinking, the eluted solution was processed using a DNA extraction kit (Bio-Dev). The specific primers used for ChIP DNA amplification are listed in S2 Table.

### Coimmunoprecipitation (Co-IP) and western blotting

Cells were lysed in radio immunoprecipitation assay (RIPA) buffer (50 mM Tris [pH 7.6], 150 mM NaCl, 2 mM EDTA, 1% Nonidet P-40, 0.1 mM PMSF) for 1 h on ice with brief vortexing every 10 min. The lysates were incubated with antibody or affinity beads as indicated overnight at 4°C. The immunoprecipitates were separated by SDS-PAGE and analyzed by immunoblotting.

### Immunofluorescence assay

At 24 h post-transfection, the cells were fixed for 30 min, blocked in goat serum, and incubated with the indicated antibodies for 1 h at RT. Cells were then washed and incubated with the appropriate secondary antibodies at 1:2500 dilution in PBS for 1 h. Slides were washed and visualized with a DM6000B fluorescence microscope (Leica, Inc.) and photographed by a digital camera and software (Leica, Inc.).

### Accession Numbers

JAG1(Gene ID: 182); RTA/ORF50 (Gene ID: 4961526); Notch1 (Gene ID:4851); Hes1 (Gene ID: 3280);RBP-Jκ(Gene ID:3516); K-bZIP/K8 (Gene ID: 4961462); vIL6 (Gene ID:4961449); ORF45 (Gene ID: 4961474); ORF59 (Gene ID: 4961492); Dll4 (Gene ID: 54567); vFLIP/ORF71 (Gene ID: 4961494); vGPCR/K14 (Gene ID: 4961465); LANA/ORF73 (Gene ID: 4961527); LEF1 (Gene ID: 51176); Hey1 (Gene ID: 23462); K5 (Gene ID: 4961442); ORF57 (Gene ID: 4961525); K3 (Gene ID: 4961486); ORF6 (Gene ID: 4961521); TK/ORF21 (Gene ID: 4961484); HIF1-α (Gene ID: 3091); TLE2 (Gene ID: 7089)

## Supporting Information

S1 Fig Expression patterns of Notch signaling components in different cell lines. (A) The expression levels of Notch components were analyzed in iSLK.RGB cells, TRE-BCBL1-RTA cells, and iSLK cells treated with or without doxycycline and in HEK293T cells transfected with RTA or control plasmids (4 μg each). N.D. represents none detectable. (B) The expression of Notch components was unchanged in SLK cells treated with or without doxycycline. The data were normalized to GAPDH expression. (C) Immunofluorescence imaging with lower magnitude which showed the overall effect of RTA in up-regulating JAG1 expression in iSLK cells treated with or without doxycycline for 24 h. The JAG1 (Green) in the cell membrane and RTA (Red) in the nucleus were labeled with the indicated primary and secondary antibodies. Scale bars represent 100 μm.
(TIF)

S2 Fig Establishment of SLK-Ctrl and SLK-RTA cell lines and gene analysis from Flow Cytometry sorted cells. (A) Expression of RTA and JAG1 in SLK-RTA and SLK-Ctrl was quantified by western blotting. (B) SLK-RTA/SLK-Ctrl and iSLK.RGB were co-cultured separately using a Transwell filter (0.45-μm pore; Corning Inc.) in a non-contacting manner for 24 h. Hes1 and Hey1 were quantified in iSLK.RGB cells by qPCR from the two groups. (C-F) KSHV latent genes vFLIP, vCyclin, and LANA were quantified between group1 and group2 (C, E) and between group3 and group4 (D, F) at both mRNA and protein level. (G) RFP positive iSLK.RGB cells were sorted by flow cytometry from co-culture groups (SLK-RTA with iSLK.RGB or SLK-Ctrl with iSLK.RGB (Right panel). SLK cells alone served as the gating control (Left panel). (H) RFP and GFP double positive iSLK.RGB cells after doxycycline induction were sorted by flow cytometry from co-culture groups (Right panel). SLK alone and iSLK.RGB without induction served as the gating control (Left panel). (I, J) SLK-Ctrl and SLK-RTA were sorted from doxycycline untreated co-culture mixture. RTA and JAG1 expression were quantified at both protein and mRNA level. (K, L) SLK-Ctrl and SLK-RTA were sorted from doxycycline treated co-culture mixture. RTA and JAG1 expression were quantified at both protein and mRNA level.
(TIF)

S3 Fig Notch pathway inhibition by LY-411575 up-regulates lytic gene expression. (A) The expression of the indicated lytic genes was quantified by qPCR in iSLK.RGB cells treated with DMSO or DAPT for 12, 24 and 48 h. (B) iSLK cells were treated with DMSO, doxycycline and doxycycline plus DATP, JAG1, Notch1 and RTA were quantified at mRNA level. (C) The efficiency of LY-411575 in ICN1 inhibition was confirmed by western blotting. (D, E) The iSLK.RGB cells were pre-treated with LY-411575 (40 μM) or DMSO for 12 h. The transcripts of the indicated lytic genes and KSHV viral genome copy number were measured by qPCR in iSLK.RGB cells treated with DMSO, doxycycline, and doxycycline plus LY-411575 (40 μM) after 36h.
(TIF)

S4 Fig Hes1 inhibits RTA promoter activity via histone modification and Hes1 was enriched in lytic gene promoters. (A, B) ChIP assays were performed on RTA promoter by using H3K4 trimethylation and H3 acetylation antibodies in HA-Hes1 and control plasmid transfection groups. (C) Primers were designed to cover the N-box or E-box Hes1 binding motifs of various lytic gene promoters. (D) ChIP assay were performed against HA-Hes1 on ORF57, ORF59 and K8 promoter. Hes1 was enriched on Hes1 binding motifs of KSHV lytic genes.
(TIF)

S5 Fig Hes1 inhibits RTA and lytic gene promoters without RTA expression. (A, B) HEK293T cells were plated into 12-well plates at 0.5 million cells per well, a dual luciferase assay was performed in HEK293T cells transiently transfected with various reporter plasmids containing RTA and lytic gene promoters (100 ng), and increasing amounts of Hes1 (250 ng, 500ng or 1 μg) using Lipofectamine 2000. Total transfected DNA was normalized with pcDNA3.1. (C, D) A dual luciferase assay was performed in HEK293T cells transiently transfected with different vector combinations. (C) Mutant K8 (Left) or mutant ORF59 (Right) promoters were transfected with increasing amounts of Hes1 (250 ng, 500 ng or 1 μg). (D) Increasing amount of mutant Hes1 or wild type Hes1 containing plasmids were transfected with K8 or ORF59 promoters. Data were expressed as the mean ± s.e.m., n = 3, *p<0.05, **p<0.01, ***p<0.001.
(TIF)

S6 Fig Reduced Hes1 restores lytic genes expression in the co-culture system. (A) Schematic illustrating the co-culture systems. iSLK.RGB cells were pre-treated with DMSO or DAPT (40 uM) for 12 h. Then SLK-Ctrl cells (0.4 million cells) or SLK-RTA cells (0.4 million cells) were co-cultured with iSLK.RGB cells (0.4 million cells) in 100 mm dish. The co-cultured cells were treated with doxycycline or doxycycline plus DAPT for 36 h before harvesting for analysis. (B) The relative lytic gene expressions in group5, 6 and 7 were detected by qPCR. (C) The expressions of ICN1 and Hes1 in co-culture systems were detected by western blotting respectively.
(TIF)

S7 Fig RTA up-regulates JAG1 expression independent of the canonical NF-κB and Notch pathways. (A) The expressions of NF-κB components were measured by western blotting in iSLK cells treated with or without doxycycline for 24 h. (B) JAG1, Hes1, and Hey1 were quantified in iSLK cells treated with DMSO, doxycycline and doxycycline plus BAY11-7082 (10 μM) for 2 h. (C) The inhibitory efficiency of BAY11-7082 against the NF-κB pathway was evaluated. TNFα treatment (100 ng/ml) activated NF-κB in iSLK cells, and this was abolished by BAY11-7082 (10 μM). (D, E) iSLK cells were pre-treated with DAPT (40 μM) or DMSO for 12 h. JAG1, Hes1 and Hey1 were quantified by western blotting (D) or qPCR (E) in iSLK cells treated with DMSO or doxycycline in the presence of newly added DMSO or DAPT for 24 h.
(TIF)

S1 Table Hes1-binding sites in KSHV lytic gene promoters. (DOCX)

S2 Table Primers for PCR amplification and qPCR analysis. RT: real-time; Restriction sites are underlined.
(DOCX)
