# Supplementary figures and images for: Fine-Tuning of the Kaposi’s Sarcoma-Associated Herpesvirus Life Cycle in Neighboring Cells through the RTA-JAG1-Notch Pathway
Source: PLoS Pathog. 2016 Oct 19;12(10):e1005900. doi: 10.1371/journal.ppat.1005900 (PMC5070770; doi:10.1371/journal.ppat.1005900)

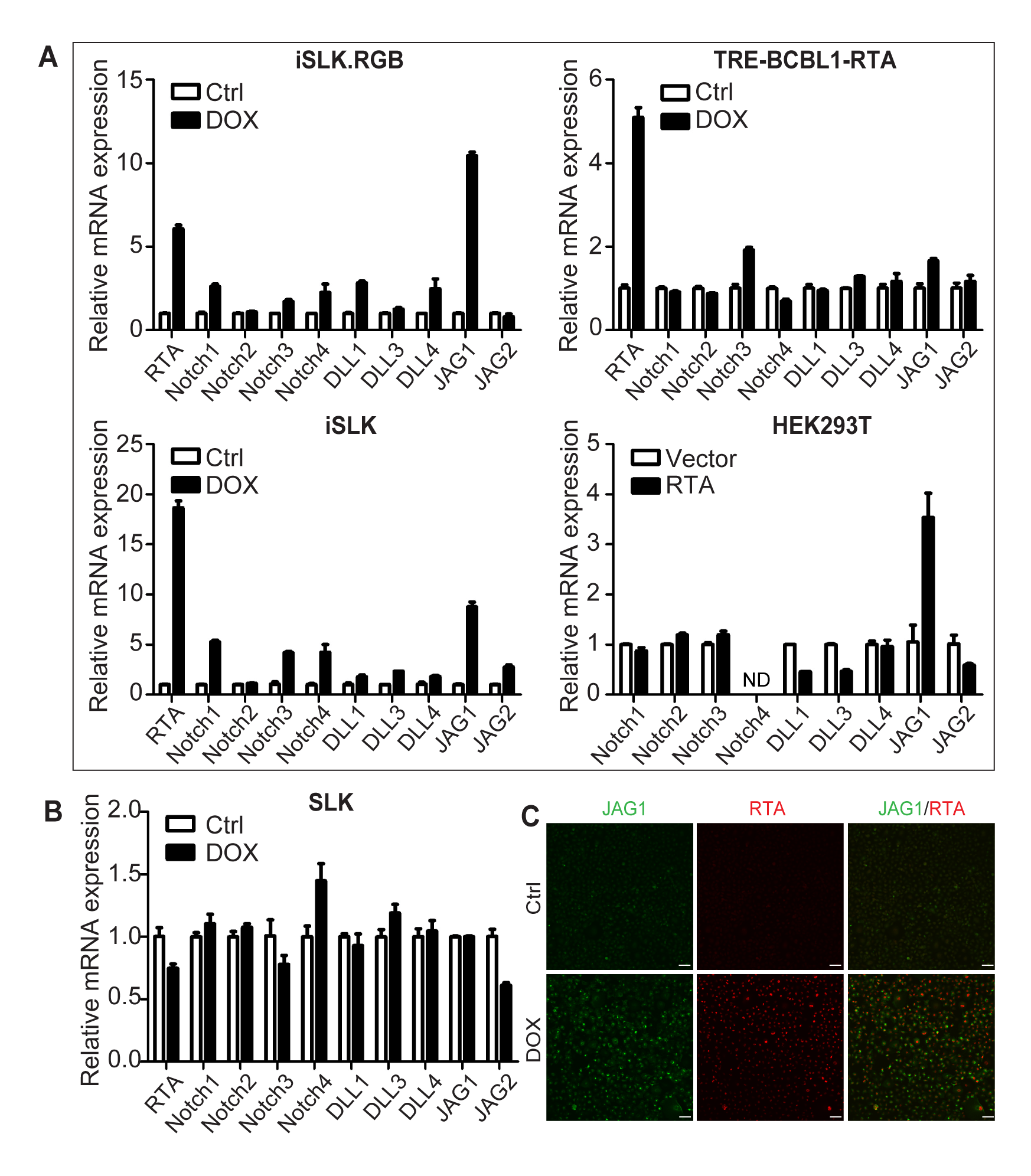

Supplement: S1 Fig — (A) The expression levels of Notch components were analyzed in iSLK.RGB cells, TRE-BCBL1-RTA cells, and iSLK cells treated with or without doxycycline and in HEK293T cells transfected with RTA or control plasmids (4 μg each). N.D. represents none detectable. (B) The expression of Notch components was unchanged in SLK cells treated with or without doxycycline. The data were normalized to GAPDH expression. (C) Immunofluorescence imaging with lower magnitude which showed the overall effect of RTA in up-regulating JAG1 expression in iSLK cells treated with or without doxycycline for 24 h. The JAG1 (Green) in the cell membrane and RTA (Red) in the nucleus were labeled with the indicated primary and secondary antibodies. Scale bars represent 100 μm. (TIF) [file ppat.1005900.s001.tif]

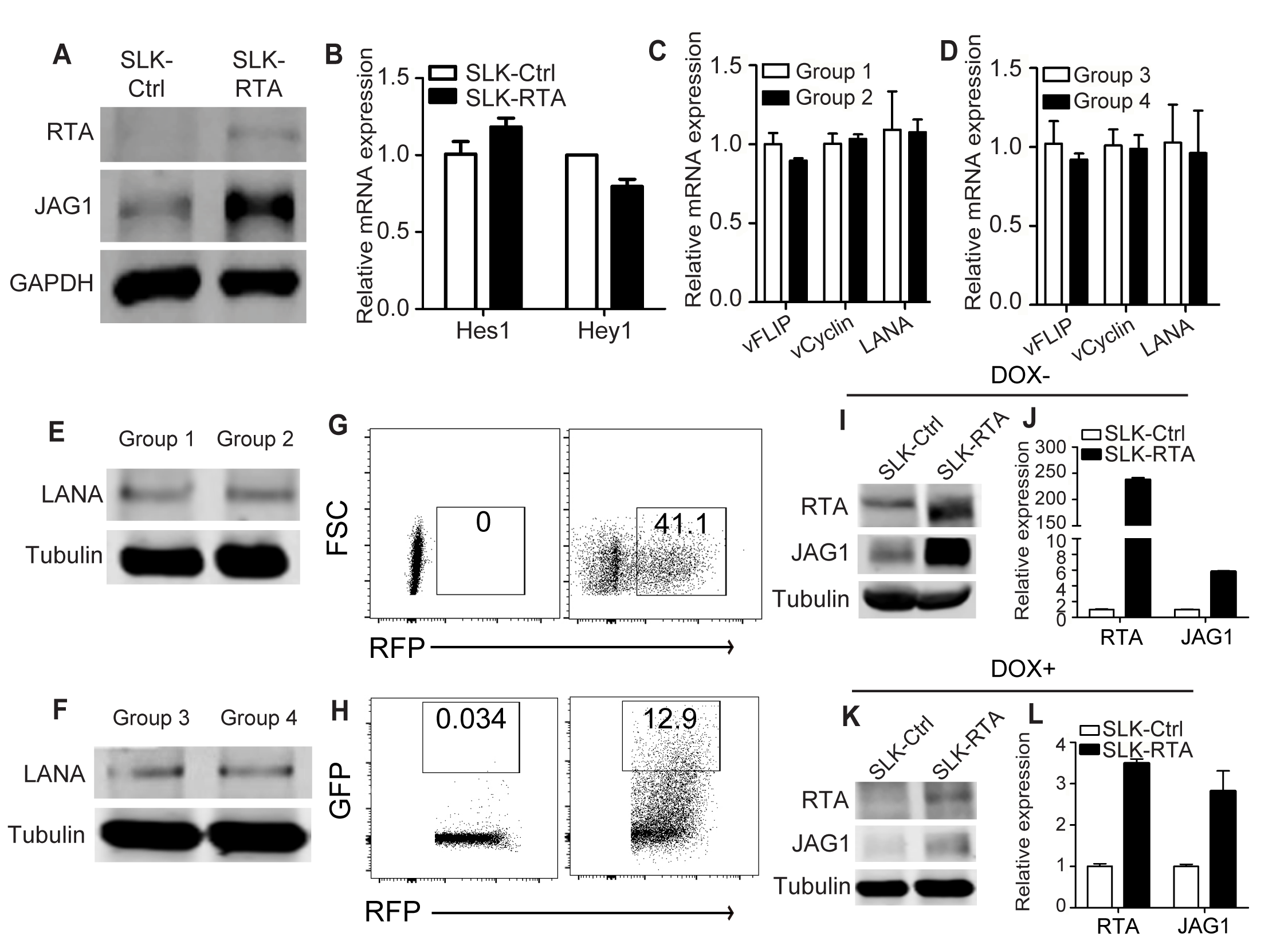

Supplement: S2 Fig — (A) Expression of RTA and JAG1 in SLK-RTA and SLK-Ctrl was quantified by western blotting. (B) SLK-RTA/SLK-Ctrl and iSLK.RGB were co-cultured separately using a Transwell filter (0.45-μm pore; Corning Inc.) in a non-contacting manner for 24 h. Hes1 and Hey1 were quantified in iSLK.RGB cells by qPCR from the two groups. (C-F) KSHV latent genes vFLIP, vCyclin, and LANA were quantified between group1 and group2 (C, E) and between group3 and group4 (D, F) at both mRNA and protein level. (G) RFP positive iSLK.RGB cells were sorted by flow cytometry from co-culture groups (SLK-RTA with iSLK.RGB or SLK-Ctrl with iSLK.RGB (Right panel). SLK cells alone served as the gating control (Left panel). (H) RFP and GFP double positive iSLK.RGB cells after doxycycline induction were sorted by flow cytometry from co-culture groups (Right panel). SLK alone and iSLK.RGB without induction served as the gating control (Left panel). (I, J) SLK-Ctrl and SLK-RTA were sorted from doxycycline untreated co-culture mixture. RTA and JAG1 expression were quantified at both protein and mRNA level. (K, L) SLK-Ctrl and SLK-RTA were sorted from doxycycline treated co-culture mixture. RTA and JAG1 expression were quantified at both protein and mRNA level. (TIF) [file ppat.1005900.s002.tif]

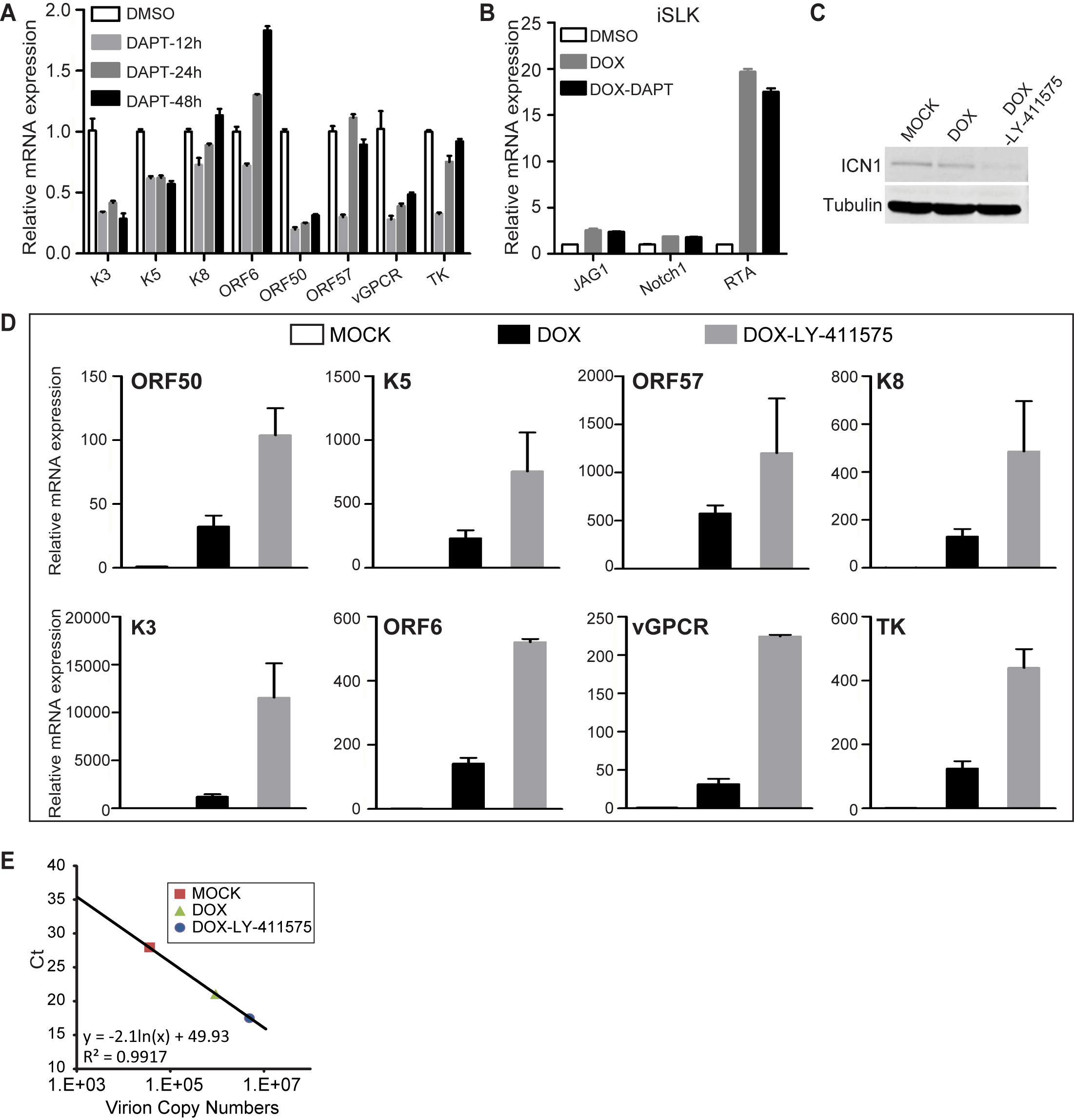

Supplement: S3 Fig — (A) The expression of the indicated lytic genes was quantified by qPCR in iSLK.RGB cells treated with DMSO or DAPT for 12, 24 and 48 h. (B) iSLK cells were treated with DMSO, doxycycline and doxycycline plus DATP, JAG1, Notch1 and RTA were quantified at mRNA level. (C) The efficiency of LY-411575 in ICN1 inhibition was confirmed by western blotting. (D, E) The iSLK.RGB cells were pre-treated with LY-411575 (40 μM) or DMSO for 12 h. The transcripts of the indicated lytic genes and KSHV viral genome copy number were measured by qPCR in iSLK.RGB cells treated with DMSO, doxycycline, and doxycycline plus LY-411575 (40 μM) after 36h. (TIF) [file ppat.1005900.s003.tif]

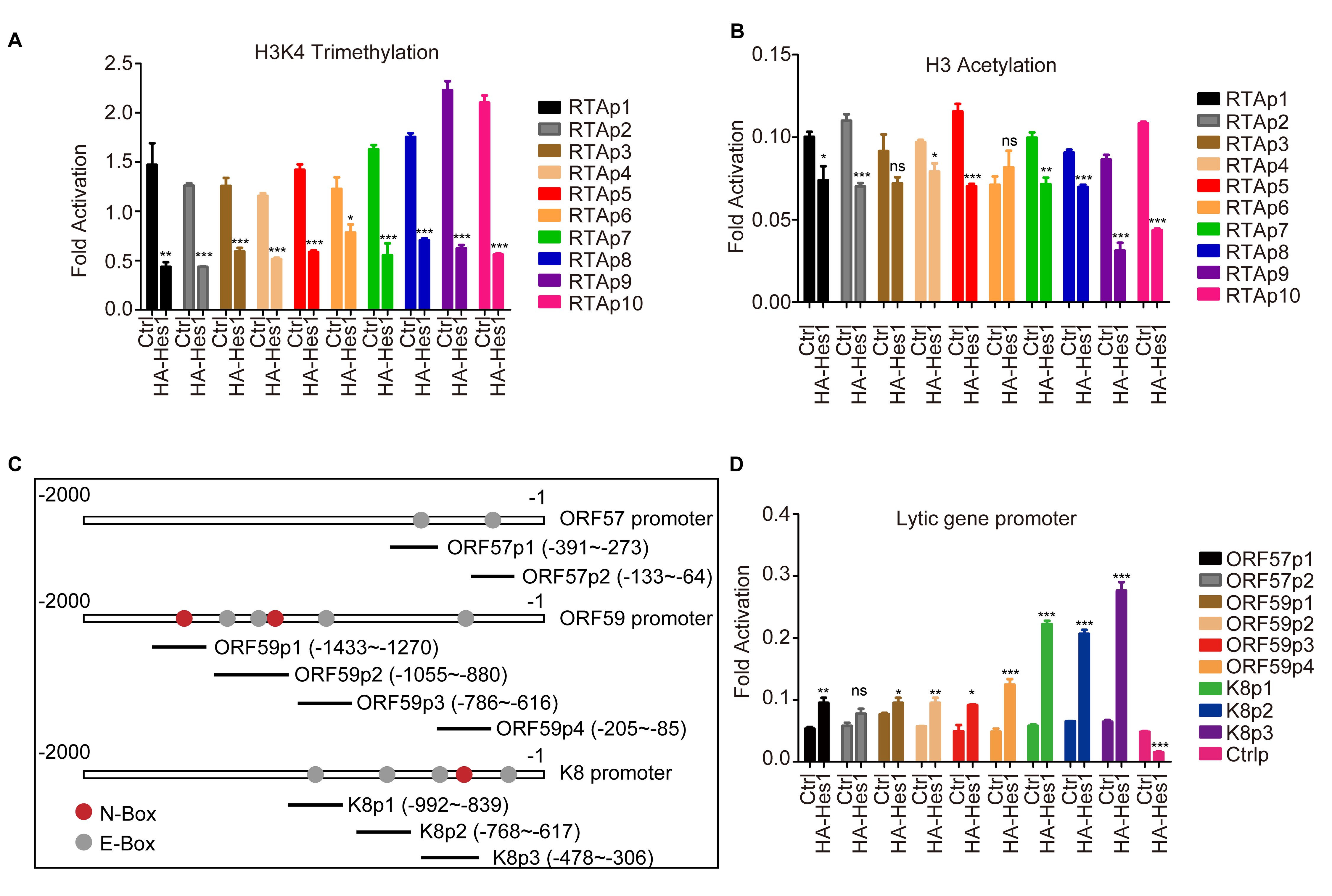

Supplement: S4 Fig — (A, B) ChIP assays were performed on RTA promoter by using H3K4 trimethylation and H3 acetylation antibodies in HA-Hes1 and control plasmid transfection groups. (C) Primers were designed to cover the N-box or E-box Hes1 binding motifs of various lytic gene promoters. (D) ChIP assay were performed against HA-Hes1 on ORF57, ORF59 and K8 promoter. Hes1 was enriched on Hes1 binding motifs of KSHV lytic genes. (TIF) [file ppat.1005900.s004.tif]

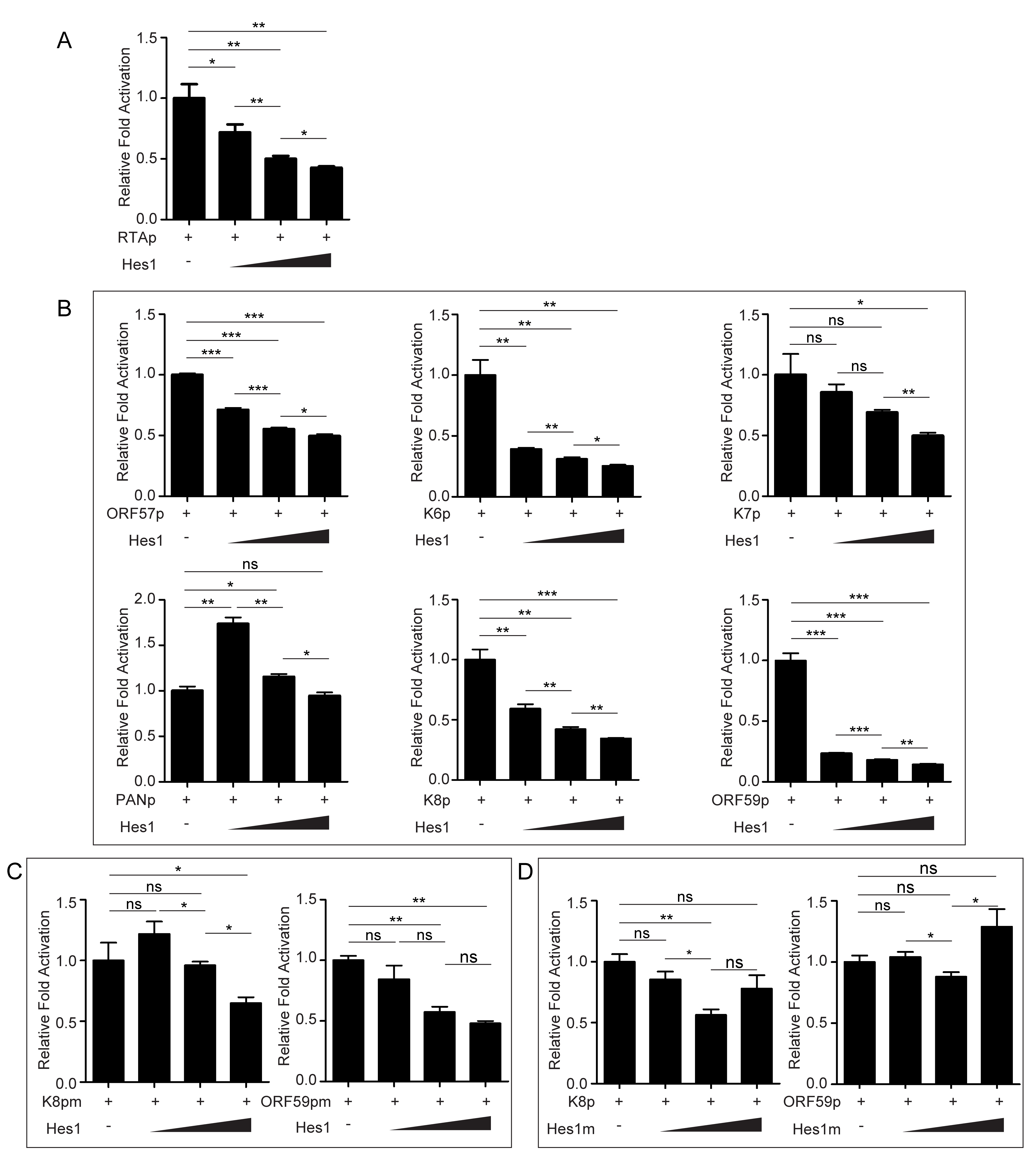

Supplement: S5 Fig — (A, B) HEK293T cells were plated into 12-well plates at 0.5 million cells per well, a dual luciferase assay was performed in HEK293T cells transiently transfected with various reporter plasmids containing RTA and lytic gene promoters (100 ng), and increasing amounts of Hes1 (250 ng, 500ng or 1 μg) using Lipofectamine 2000. Total transfected DNA was normalized with pcDNA3.1. (C, D) A dual luciferase assay was performed in HEK293T cells transiently transfected with different vector combinations. (C) Mutant K8 (Left) or mutant ORF59 (Right) promoters were transfected with increasing amounts of Hes1 (250 ng, 500 ng or 1 μg). (D) Increasing amount of mutant Hes1 or wild type Hes1 containing plasmids were transfected with K8 or ORF59 promoters. Data were expressed as the mean ± s.e.m., n = 3, *p<0.05, **p<0.01, ***p<0.001. (TIF) [file ppat.1005900.s005.tif]

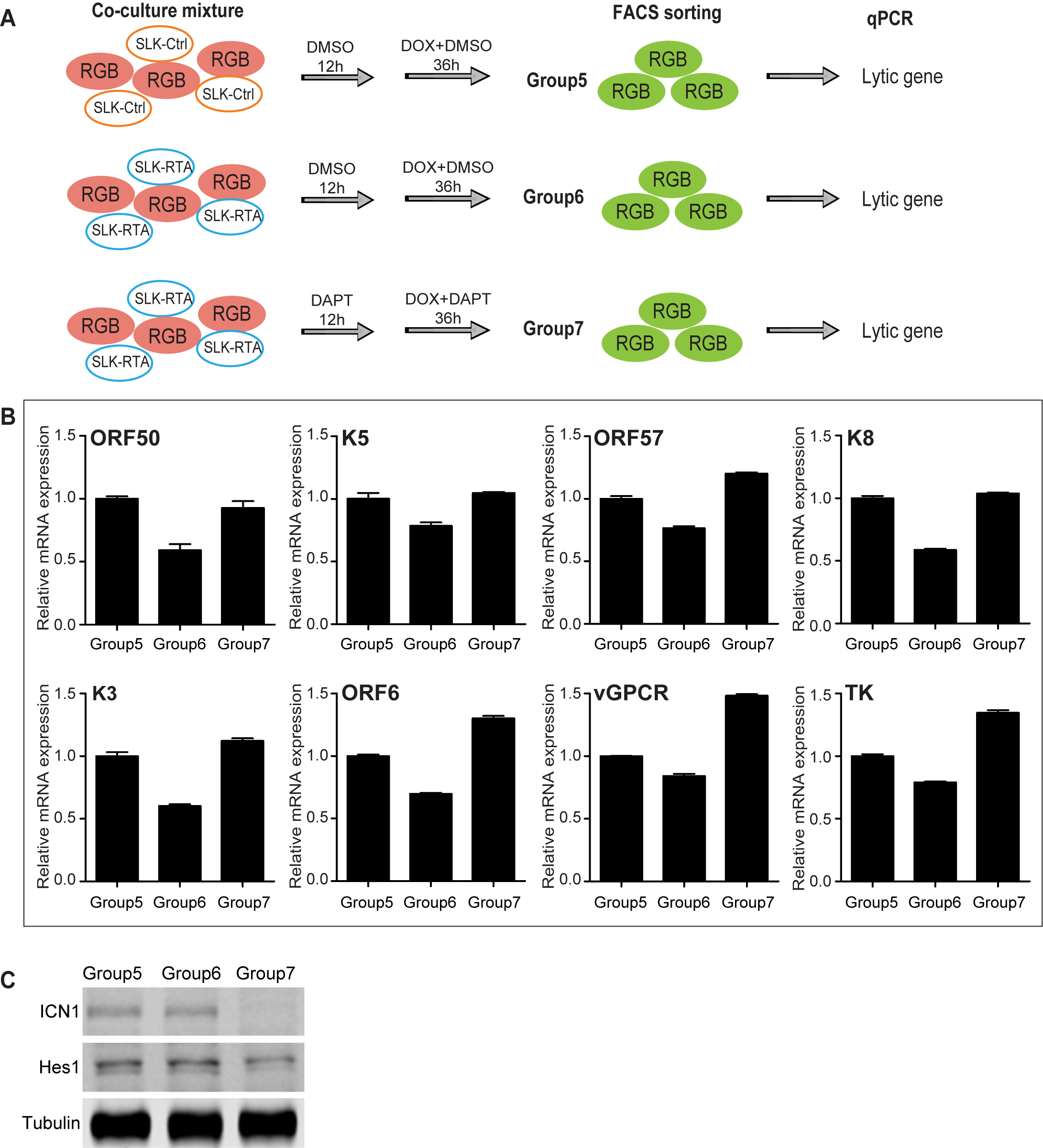

Supplement: S6 Fig — (A) Schematic illustrating the co-culture systems. iSLK.RGB cells were pre-treated with DMSO or DAPT (40 uM) for 12 h. Then SLK-Ctrl cells (0.4 million cells) or SLK-RTA cells (0.4 million cells) were co-cultured with iSLK.RGB cells (0.4 million cells) in 100 mm dish. The co-cultured cells were treated with doxycycline or doxycycline plus DAPT for 36 h before harvesting for analysis. (B) The relative lytic gene expressions in group5, 6 and 7 were detected by qPCR. (C) The expressions of ICN1 and Hes1 in co-culture systems were detected by western blotting respectively. (TIF) [file ppat.1005900.s006.tif]

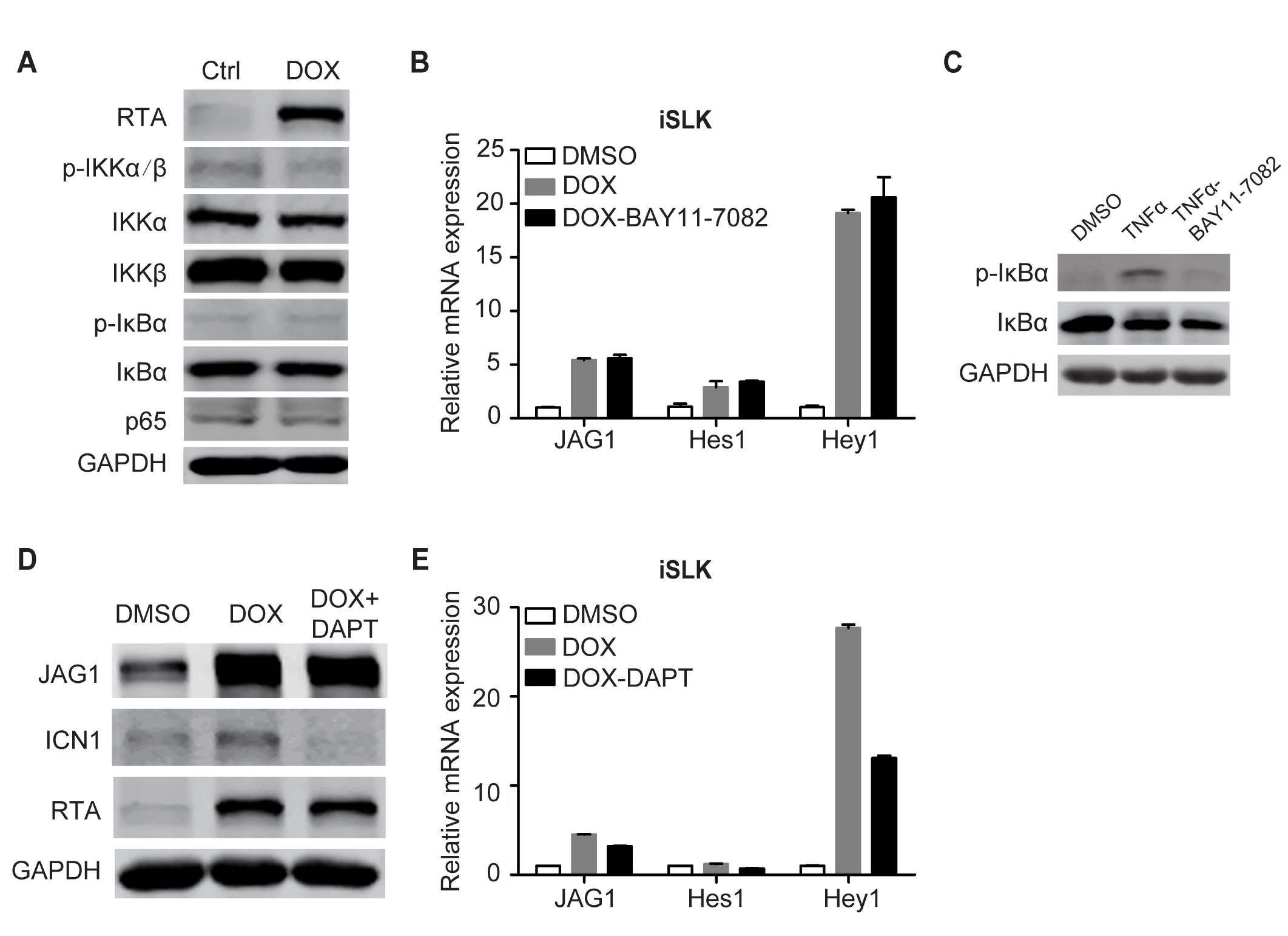

Supplement: S7 Fig — (A) The expressions of NF-κB components were measured by western blotting in iSLK cells treated with or without doxycycline for 24 h. (B) JAG1, Hes1, and Hey1 were quantified in iSLK cells treated with DMSO, doxycycline and doxycycline plus BAY11-7082 (10 μM) for 2 h. (C) The inhibitory efficiency of BAY11-7082 against the NF-κB pathway was evaluated. TNFα treatment (100 ng/ml) activated NF-κB in iSLK cells, and this was abolished by BAY11-7082 (10 μM). (D, E) iSLK cells were pre-treated with DAPT (40 μM) or DMSO for 12 h. JAG1, Hes1 and Hey1 were quantified by western blotting (D) or qPCR (E) in iSLK cells treated with DMSO or doxycycline in the presence of newly added DMSO or DAPT for 24 h. (TIF) [file ppat.1005900.s007.tif]
