# Supplementary material for: Fine-Tuning of the Kaposi’s Sarcoma-Associated Herpesvirus Life Cycle in Neighboring Cells through the RTA-JAG1-Notch Pathway
Source: PLoS Pathog. 2016 Oct 19;12(10):e1005900. doi: 10.1371/journal.ppat.1005900 (PMC5070770; doi:10.1371/journal.ppat.1005900)
Supplement: S1 Table — (DOCX) [file ppat.1005900.s008.docx]

**S1 Table. Hes1-binding sites in KSHV lytic gene promoters.**

| **Gene** | **N-box** | **E-box** |
| --- | --- | --- |
| PAN | 0 | 0 |
| ORF K7 | 0 | 2 |
| ORF57 | 0 | 2 |
| ORF K6 | 0 | 4 |
| ORF K8 | 1 | 5 |
| ORF59 | 2 | 4 |
| ORF50 | 3 | 4 |
