# Supplementary material for: Fine-Tuning of the Kaposi’s Sarcoma-Associated Herpesvirus Life Cycle in Neighboring Cells through the RTA-JAG1-Notch Pathway
Source: PLoS Pathog. 2016 Oct 19;12(10):e1005900. doi: 10.1371/journal.ppat.1005900 (PMC5070770; doi:10.1371/journal.ppat.1005900)
Supplement: S2 Table — RT: real-time; Restriction sites are underlined. (DOCX) [file ppat.1005900.s009.docx]

**S2 Table. Primers for PCR amplification and qPCR analysis.**

| **Primer Name** | **Sequence (5’ - 3’)** |
| --- | --- |
| RTA-F | TAGTGAACCGTCAGATCGCCCTCGAGATGGCGCAAGATGACAAGGGTAAGA |
| RTA-R | CCGGAATTCGTCTCGGAAGTAATTACGCCATTGG |
| Hes1-F | CCGGAATTCACATGCCAGCTGATATAATGGAG |
| Hes1-R | CGCCGCCTCGAGTCAGTTCCGCCACGGCCTCCACA |
| Hes1m-F | CCG*GAATTC*ACATGCCAGCTGATATAATGGAG |
| Hes1m-R | CGCCGC*CTCGAG*TCAGTTCCGCCCCGGCCTCCCCATGGAGTCCGC |
| LEF1-F | CCGGAATTCAAATGCCCCAACTCTCCGGA |
| LEF1-R | CCGGGTACCTCAGATGTAGGCAGCTGTCATTC |
| RT-notch1-F | GAGGCGTGGCAGACTATGC |
| RT-notch1-R | CTTGTACTCCGTCAGCGTGA |
| RT-JAG1-F | GTCCATGCAGAACGTGAACG |
| RT-JAG1-R | GCGGGACTGATACTCCTTGA |
| RT-Hes1-F | TACTTCCCCAGCACACTTGG |
| RT-Hes1-R | CGGACATTCTGGAAATGACA |
| RT-Hey1-F | CGAAATCCCAAACTCCGATA |
| RT-Hey1-R | TGGATCACCTGAAAATGCTG |
| RT-ORF50-F | CACAAAAATGGCGCAAGATGA |
| RT-ORF50-R | TGGTAGAGTTGGGCCTTCAGTT |
| RT-K5-F | ACAAGGACCGTCAATTCGATG |
| RT- K5-R | TGCCATACCGACGGCC |
| RT-ORF57-F | TGGACATTATGAAGGGCATCCTA |
| RT-ORF57-R | CGGGTTCGGACAATTGCT |
| RT-K8-F | CATGCTGATGCGAATGTGC |
| RT-K8-R | AGCTTCAACATGGTGGGAGTG |
| RT-K3-F | AGCCCCATCGCCCG |
| RT-K3-R | TGAGCGGTATAGGGCCACTTAC |
| RT-ORF6-F | CTGCCATAGGAGGGATGTTTG |
| RT-ORF6-R | CCATGAGCATTGCTCTGGCT |
| RT-vGPCR-F | TGGTGGGCCTATTTGGGATA |
| RT-vGPCR-R | GATGCACCG CCCTGCTT |
| RT-TK-F | CGTAGCCGACGCGGATAA |
| RT-TK-R | TGCCTGTAGATTTCGGTCCAC |
| RT-K9-F | GTCTCTGCGCCATTCAAAAC |
| RT-K9-R | CCGGACACGACAACTAAGAA |
| RT-3’UTR-ORF50-F | ACGGGAAACAGGTGTCTATCTT |
| RT-3’UTR-ORF50-R | GTTCCTTATGTGCCTCCAATCT |
| K6p-F | CTACTAGCTAGCAATCACTATGTTGGGTCACCC |
| K6p-R | CGACCCAAGCTTCTGGAGGTGCCAAGTTCGC |
| K7p-F | CTACTAGCTAGCCAAAAGTGATTGTTGGGGCG |
| K7p-R | CGACCCAAGCTTTTTTGGACAGGTTAGCCACC |
| ORF59p-F | CGGGTACCTCGATGCTGAGAGC |
| ORF59p-R | ATAGATCTGATTGCGGCCGTAGACGCAC |
| K8pm-F | TATACTCTCAAGGTGACGGTATATCCGC |
| K8mp-R | CGTCACCTTGAGAGTATACGCAACTGCAAC |
| ORF59mp-F | CTACTAGCTAGCAGCGATGCCGACAAGGAC |
| ORF59mp-R | ATAGATCTGATTGCGGCCGTAGACGCAC |
| CHIP-Ctrl-F | CTGATATCCAGGAGGAGAAAGG |
| CHIP-Ctrl-R | AGCCCGACAATGTCAAGGACTG |
| CHIP-Hes1-RTAp-F | GCAAGCTGGTGTTCTGGATT |
| CHIP-Hes1-RTAp-R | CCTGGAAAGAACTGTCGGTAA |
| CHIP-RTAp-F1 | TCACCAGGGTATCCAAAAGC |
| CHIP-RTAp-R1 | TGGAAGAGTATGGCGGACTG |
| CHIP-RTAp-F2 | CAGTCCGCCATACTCTTCCA |
| CHIP-RTAp-R2 | GCTTAGCTTGATTTCATCGTGT |
| CHIP-RTAp-F3 | TCCGAGGTAATGTGCTCTAT |
| CHIP-RTAp-R3 | TTTTCTGAGTTTTCTATTCCAG |
| CHIP-RTAp-F4 | AAAGACACTGACCCACCAAG |
| CHIP-RTAp-R4 | AGAAATCACCCACTGTAGCG |
| CHIP-RTAp-F5 | GATGAGCCCCGCAGAAAGAA |
| CHIP-RTAp-R5 | GATAGATTTATGCTGATACTGGAA |
| CHIP-RTAp-F6 | TCTACCACGGTCATACATTG |
| CHIP-RTAp-R6 | AAATATGTGGGACAGGAAAT |
| CHIP-RTAp-F7 | TGCAGTCATCCCAGATCAAA |
| CHIP-RTAp-R7 | TTCTTAGCGTCCCTGGTGGT |
| CHIP-RTAp-F8 | AAGTAGATTCAGTCTGGTGTTG |
| CHIP-RTAp-R8 | AAGACCGAGCGTATTCTCAG |
| CHIP-RTAp-F9 | GAATACGCTCGGTCTTGACG |
| CHIP-RTAp-R9 | ATGTGACTGAACATGGAGGTGT |
| CHIP-RTAp-F10 | GTACCGAATGCCACAATCTG |
| CHIP-RTAp-R10 | AGCTGTAGCTGGGTCCTATG |
| CHIP-RTAp-F11 | GCTACAGCTTATCCTCCACTAAA |
| CHIP-RTAp-R11 | CTGCCTGGACAGTATTCTCAC |
| K8-ChIP-1-F | ATGCGTTACGTTGTTGCAGGTTACC |
| K8-ChIP-1-R | TCGCCCGAGGCTTCTGGTTG |
| K8-ChIP-2-F | CAGAGGCGACAACACCCAAACG |
| K8-ChIP-2-R | GCACATCCTGGGGGGTGGTAA |
| K8-ChIP-3-F | CCTCTCTCCCCCACTGACGACG |
| K8-ChIP-3-R | CCTGACCGGTTGCAGTTGCGTAT |
| 57-ChIP-1-F | CCGGTCGTTCAATAGAGGACTGG |
| 57-ChIP-1-R | GCGTAAATGCAACGTGAGAAAACTG |
| 57-ChIP-2-F | CACTTATGAGTCAGTGTTTTGCCAG |
| 57-ChIP-2-R | CACAAACGAAAAATGGGCCG |
| 59-ChIP-1-F | AGGGTATTCCTAACAGCCTTTGATT |
| 59-ChIP-1-R | GTATCCATGTAGCAGCAGGTCCC |
| 59-ChIP-2-F | GAGGCCAATGGATTCAGTTGA |
| 59-ChIP-2-R | AAAAATCTATAAAAGTCCTTGTCGG |
| 59-ChIP-3-F | CCGAACAGGTTGCCATGGAGAATAT |
| 59-ChIP-3-R | GGTCGCCTTACGCACCCTGGA |
| 59-ChIP-4-F | GGCAGTTTCAAGGCTGTGAATTTTT |
| 59-ChIP-4-R | ACTTTTAGGGGAGGTGGAAGTGTGC |
